# Theoretical study on the design of allosteric inhibitors of diabetes associated protein PTP1B

**DOI:** 10.3389/fphar.2024.1423029

**Published:** 2024-08-22

**Authors:** Jiuyu Zhan, Zhenyang Liu, Hongwei Gao

**Affiliations:** School of Life Science, Ludong University, Yantai, Shandong, China

**Keywords:** protein tyrosine phosphatase 1B, inhibitor, MD simulations, rational design, LUDI

## Abstract

The protein tyrosine phosphatase 1B (PTP1B) is a critical therapeutic target for type 2 diabetes mellitus (T2DM). Many PTP1B inhibitors have been reported, however, most of them lack high specificity and have adverse effects. Designing effective PTP1B inhibitors requires understanding the molecular mechanism of action between inhibitors and PTP1B. To this end, molecular dynamics (MD) simulations and molecular mechanics Poisson Boltzmann Surface Area (MM-PB/SA) methods were used to observe the binding patterns of compounds with similar pentacyclic triterpene parent ring structures but different inhibition abilities. Through structure and energy analysis, we found that the positions of cavities and substituents significantly affect combining capacity. Besides, we constructed a series of potential inhibitor molecules using LUDI and rational drug design methods. The ADMET module of Discovery Studio 2020 was used to predict the properties of these inhibitor molecules. Lastly, we obtained compounds with low toxicity and significant inhibitory activity. The study will contribute to the treatment of T2DM.

## 1 Introduction

Diabetes mellitus (DM) is a common systemic disease of the endocrine system (Ma et al., 2021; Singh et al., 2022). This major public health issue and socioeconomic burden endangers human health worldwide (Hünenberger et al., 1995; Lam and LeRoith, 2012). In 2045, there will be 220 million people living with diabetes worldwide, according to the International Diabetes Federation (Paul et al., 2023). A majority of people with diabetes are aware of type 2 diabetes, which represents 90%–95% of diabetes cases. Insulin resistance (IR) and relative insulin deficiency are characteristics of diabetes mellitus type 2 (T2DM) (Verma et al., 2017; Jiang and Gao, 2019).

The proteins of protein tyrosine phosphatases (PTP) family are a class of phosphatases that play an essential role in signal transduction pathways that regulate the progression of cell growth, division, adhesion, and motility (Hunter, 1995; Hunter, 2000). Disruption of PTP catalytic activity will lead to abnormal tyrosine phosphorylation, resulting in the development and progression of various diseases (Peters et al., 2003). Protein tyrosine phosphatase 1B (PTP1B), an essential member of the PTP family, is a negative regulator of insulin receptor (IR) signaling that negatively regulates insulin signaling through dephosphorylation of the insulin receptor and its substrates, thereby diminishing the effect of insulin (Combs, 2010; Lessard et al., 2010). In addition, PTP1B knockout mice exhibit insulin sensitivity and glycemic control, are resistant to obesity, and have significantly lower triglycerides levels (Elchebly et al., 1999; Bence et al., 2006; Comeau et al., 2010). Hence, PTP1B has emerged as a novel promising therapeutic target for the treatment of T2DM.

Protein tyrosine phosphatases have high structural conserved properties at the active site, which makes the design and modification of selective PTP1B inhibitors very difficult (Andersen et al., 2001). TCPTP and PTP1B showed 72% sequence identity in the catalytic region, while mice knocked out by TCPTP showed hematopoietic defects (Shinde and Sobhia, 2013). Therefore, competitive inhibitors targeting the catalytic site of PTP1B may also bind to the catalytic site of TCPTP, causing hematopoietic dysfunction. Compared with competing inhibitors, PTP1B allosteric inhibitors have low side effects and do not cause PTP1B aggregation (Krishnan et al., 2014; Krishnan and Tonks, 2015; Krishnan et al., 2018). At the preclinical level, trodusquemine, an allosteric inhibitor of PTP1B, demonstrated the ability to significantly reduce fat and insulin levels in obese mice (Lantz et al., 2010; Cho and Litwack, 2013; Olloquequi et al., 2022), which means that allosteric inhibitors can exert inhibitory activities that are no less potent than competitive inhibitors. Therefore, the design of novel allosteric inhibitors can be considered.

PTP1B has an N-terminal catalytic phosphatase domain, as well as a C-terminal membrane localization domain (Tonks et al., 1988). The active site of PTP1B consists of three parts: (1) P-loop with Cys215 as the catalytic center; (2) WPD-loop responsible for substrate identification; (3) Q-loop containing Gln262 residues (Tonks, 2003). The closed pose of the WPD ring is the result of the interaction between the α3 (Glu186-Glu200) and α6 (Ala264-Ile279) helixes (Shinde and Sobhia, 2013). The active site actually includes α7(Val287-Ser295), but α7 unspins when it binds to an allosteric inhibitors (Wiesmann et al., 2004a). While the movement of the WPD ring is the result of the hydrogen-bond network among α7-α3-α6 helixes. While, allosteric inhibitors can destroy this hydrogen-bond network by placing themselves among these helixes, thereby blocking the open or closed conformation of the PTP1B protein, rendering it unable to function. To this, it is important to discover novel PTP1B inhibitors with good inhibitory activity and selectivity.

Currently, the majority of studies are centered on the movement pattern of PTP1B function and the search for inhibitors of PTP1B. Regrettably, these literatures carried their own limitations. For instance, in the articles investigating the movement process, collaboration, and interaction of PTP1B through molecular dynamics, the majority failed to present viewpoints on inhibiting the activity of PTP1B (Akyol and Kilic, 2021), while the literatures devoted to finding or designing inhibitors of PTP1B did not undertake subsequent exploration of the action mode for the discovered inhibitors (Maccari et al., 2023; Zheng et al., 2024).

In the present study, molecular dynamics (MD) simulations combined with molecular mechanics Poisson Boltzmann Surface Area (MM-PB/SA) calculations, which have proved to be robust and valuable tools (Pan et al., 2016; Coskuner and Uversky, 2017; Wen et al., 2017; Shi et al., 2018; Wang et al., 2018; Alamri et al., 2023; Gao et al., 2023; Hassan et al., 2023), were used to explore the interaction and binding capacity between inhibitors and PTP1B. AMDET property prediction is used to evaluate compounds’ molecular properties and select and optimize lead compounds according to their properties. The parent structures with analogous configurations were exploited to investigate the binding action mode and motion mode between the intermolecular inhibitors and PTP1B. Based on this, drug design strategies were proposed. Eventually, the feasibility of our results was verified via the analysis of binding free energy and druggability. Our work may provide valuable clues for drug modification and improve binding affinity to combat drug resistance.

## 2 Materials and methods

### 2.1 Initial structures

**FIGURE 1:**
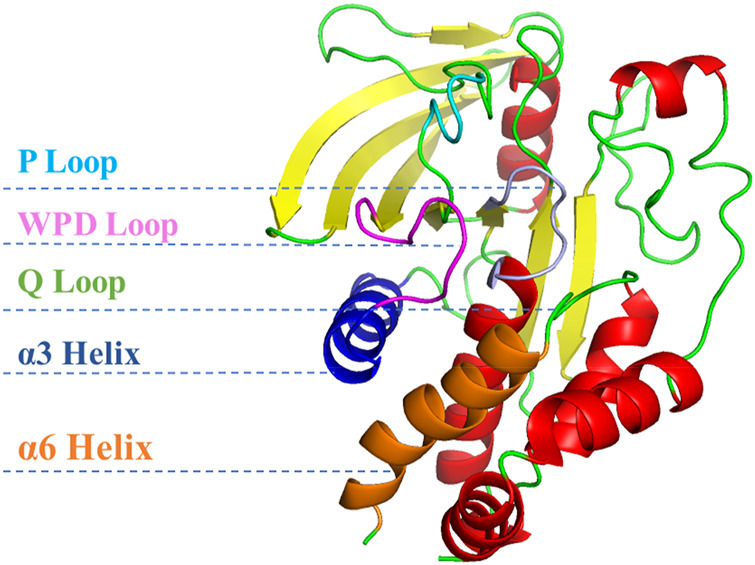
Structure of PTP1B protein (PDB ID: 1T49).

**FIGURE 2:**
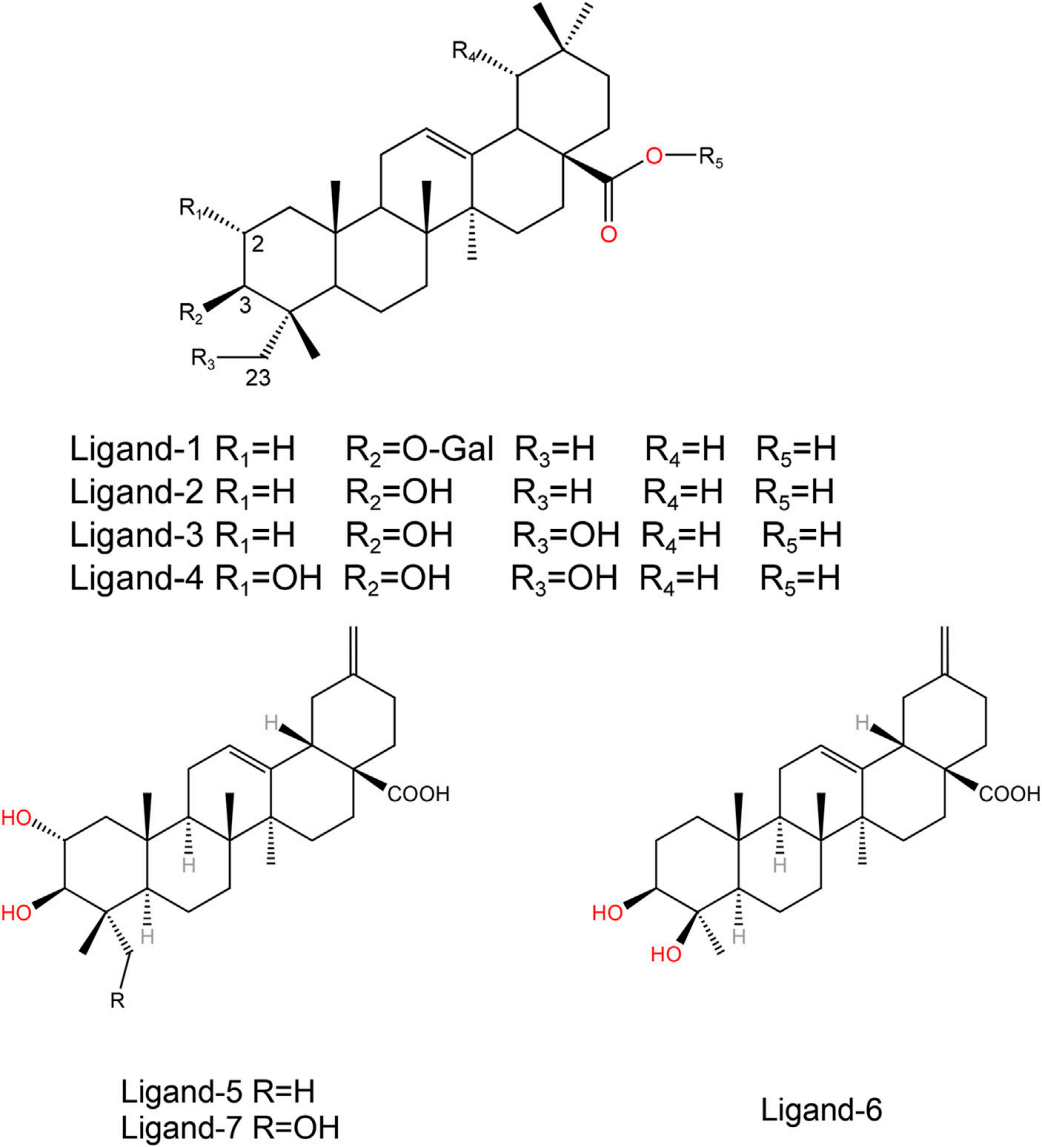
Structures of the ligand compounds.

The initial structure of PTP1B protein was derived from Protein Data Bank (PDB code: 1T49) (Figure 1) (Wiesmann et al., 2004b). Compared with other PDB structures(7KEN, 5T19, 1T48, or 5QDE), 1T49 has relatively high resolution (1.9 Å), mutation-free amino acid sequence, and the binding ligand in the crystal structure is similar to those in the present study (Jiang et al., 2022). Protein pretreatment and energy minimization of small molecules are performed by Discovery Studio 2020 (DS 2020). The position of the ligand of the original PDB crystal structure is used as the binding site. The ligands used in this article were all pentacyclic triterpenoids reported in the previous literature (Figure 2) (Tables 1, 2) (Xu et al., 2018; Huang et al., 2022). These studies have experimentally identified several compounds with inhibitory activity against PTP1B in *Quercus liaotungensis*, *Paeonia suffruticosa*, and *Paeonia delavayi*. The majority of these compounds are pentacyclic triterpenoids. Compounds with different IC_50_ values were selected to compare the structure-activity relationships of different residues. These compounds possess similar structures yet distinct substituents. Following molecular dynamics, the interaction between the compounds and the protein can be delineated more lucidly. Upon discovering these compounds, the existing literature did not provide information on the type of inhibitors. We individually docked these compounds to the catalytic and allosteric sites of PTP1B.DS2020 was used for molecular docking to dock Ligand 1-7 to specific sites on the PTP1B protein. After docking, we named these docking systems Complex1-7 based on the names of the docked ligands, for example, the ligand name in the complex-1 system is Ligand-1. The protonation states of ionizable residues were determined at pH = 7.4 using H++ server (Gordon et al., 2005). The partial charges and missing force field parameters for inhibitors were obtained by the Antechamber module of AMBER 18 software (Wang et al., 2006). The general AMBER force field (GAFF) (Wang et al., 2004) and ff14SB force field (Maier et al., 2015) were used for inhibitors and PTP1B, respectively, just as the methods employed by Lima Silva and Ferreira de Freitas (2023); Park et al. (2023). The missing atoms of proteins in the models were added using the t-Leap module of AMBER 18. To keep the whole system in an electric neutral state, sodium ions were added based on a Coulomb potential grid using t-Leap module of AMBER 18 software (Case et al., 2017). Then, each system was solvated with the TIP3P water model (Jorgensen et al., 1983) in a truncated octahedron box with a 10.0 Å distance around the solute.

**TABLE 1:** The name of the ligand compound.

NO.	Name
Ligand-1	3-O-galloyloleanolic acid
Ligand-2	oleanolic acid
Ligand-3	23-dihydroxyolean-12-en-28-oic acid
Ligand-4	arjunolic acid
Ligand-5	akebonic acid
Ligand-6	2*α*,3*β*-dihydroxy-30-noroleana-12,20(29)-dien- 28-oic acid
Ligand-7	paeonenoide C

**TABLE 2:** PTP1B inhibition of ligand compounds.

NO.	PTP1B IC_50_(*μ*M)	Ref
Ligand-1	2.10	Xu et al. (2018)
Ligand-2	17.25	Xu et al. (2018)
Ligand-3	47.60	Xu et al. (2018)
Ligand-4	>100	Xu et al. (2018)
Ligand-5	36.5	Huang et al. (2022)
Ligand-6	**–**	Huang et al. (2022)
Ligand-7	110.2	Huang et al. (2022)

### 2.2 Molecular dynamics (MD) simulations

AMBER18 software package (Case et al., 2017) was used for molecular dynamics simulation. First, 10,000 steps of minimization (steepest 4,000 steps, followed by 6,000 steps of conjugation gradient) with proteins and inhibitors constrained (500 kcal mol^-1^ Å^-2^). Then, the minimization is repeated without any constraints. Thereafter, each system was gradually heated from 0 K–310 K over a period of 300 ps with 5.0 kcal mol^-1^ Å^-2^ restrain on the solute and then another 1 ns equilibrium simulation was followed at 310 K with 2.0 kcal mol^-1^ Å^-2^ restrain on the solute. Finally, 200 ns MD simulations were performed for every system under NPT conditions to obtain the simulated trajectories. The temperature was maintained at 310 K by coupling to a Langevin heatbath (Uberuaga et al., 2004) using a collision frequency of 1 ps^-1^, and a constant isotropic pressure was maintained at 1 atm using the Berendsen barostat (Berendsen et al., 1984). Short range interactions were cut off at 10.0 Å, while the long-range electrostatic interactions were handled using the particle mesh Ewald (PME) method (Darden et al., 1993). The SHAKE algorithm was used to restrict all covalent bonds involving hydrogen atoms (Ryckaert et al., 1977). The time step was set to 2 fs.

### 2.3 MM-PB/SA calculations

Binding free energy of each complex was calculated by MM-PB/SA (Kollman et al., 2000; Sun et al., 2014; Kong et al., 2018; Federico et al., 2021) method in AMBER 18. In our calculation, the last 10,000 snapshots are extracted from each simulated trajectory to calculate the binding free energy. The equations are as follows:

$$∆G_{\text{bind}}=G_{\text{complex}}-\left(G_{\text{receptor}}+G_{\text{ligand}}\right)$$ (1)

$$∆G_{\text{bind}}=∆E_{\text{MM}}+∆G_{\text{sol}}-T∆S$$ (2)

$$∆E_{\text{MM}}=∆E_{\mathrm{int}}+∆E_{\text{ele}}+∆E_{\text{vdW}}$$ (3)

$${∆G}_{\text{sol}}{=∆G}_{\text{GB}}+∆G_{\text{SA}}$$ (4)

In Equation 1, G_complex_, G_receptor_, and G_ligand_ are the free energies of the complex, the receptor, and ligands, respectively. In Equation 2, the ∆E_MM_, ∆G_sol_, and T∆S represent molecular mechanics component in the gas phase, the desolvation free energy, and a vibrational entropy term, respectively. And in Equation 3, ∆E_MM_ is the summation of internal interaction (∆E_int_), Coulomb interaction (∆E_ele_), and van der Waals (vdW) interaction (∆E_vdW_) terms. In Equation 4, G_sol_ can be separated into an electrostatic solvation energy (∆G_GB_) and nonelectrostatic solvation energy (∆G_SA_). For obtaining the detailed view of protein and ligands interaction, MM-PB/SA method was employed to calculate the binding free energy of each residue. We selected the stable trajectory after MD simulation to calculate entropy.

### 2.4 Structure-based inhibitor design

LUDI (Böhm, 1994) is a fragment-based *de novo* drug design algorithm. In this present study, we used the pentacyclic triterpene parent structure as a starting point in this study, with amino acids from the α3 and α6 helical regions of PTP1B acting as receptor action regions, and the LUDI module in DS2020 added design fragments to the drug structure (Oner et al., 2023). In addition, we also conduct rational drug design based on the intermolecular interactions between inhibitors and amino acid residues.

### 2.5 ADMET properties prediction

ADMET properties refer to the absorption, distribution, metabolism, excretion, and toxicity of molecules in the organic body (Dong et al., 2018). Predictable properties of ADMET include water solubility, blood-brain barrier penetration (BBB), hepatotoxicity, human intestinal absorption (HIA), aqueous solubility (LogSw), and plasma protein binding. In this experiment, we applied LUDI modification and rational design compounds to ADMET prediction on DS 2020.

### 2.6 TOPKAT and druggability analysis

TOPKAT is based on the 2D structure of the molecule to calculate and verify the toxicity and environmental effects of the compound (Ma et al., 2008). The rat oral LD50 for all ligands was calculated and measured by the TOPKAT module in DS 2020. Druggability Analysis is run through DrugFlow (www.drugfow.com).

## 3 Results and discussion

### 3.1 Stability of ligands in MD simulations

The structural stability was investigated by calculating average root-mean-square deviation (RMSD) of backbone atoms with respect to the first frame. Details of these complexes systems are shown in Supplementary Figure S1.

Supplementary Figure S1 shows that the fluctuation range of RMSD of these complexes is 0.5–1.5 Å in the first 100 ns and stabilizes at about 1 Å in the last 100 ns. In the whole simulation process, all the complex structures are relatively stable. Therefore, for all systems, the last 100 ns trajectories are selected for further analyses.

To analyze the variation of flexibility in seven complexes, average root-mean-square fluctuations (RMSF) of backbone atoms were calculated. The RMSF of the corresponding complexes systems was calculated. By comparing the unbound complex with the one bound to a natural PTP1B inhibitor, it is evident that the fluctuation of the WPD-loop (Thr177-Pro185) in the protein without ligands is more pronounced, reaching approximately 2.0 Å (Supplementary Figure S5), this result indicates that the protein without ligands is indeed less stable. According to the literature, the movement of the allosteric site can influence the conformational dynamics of the WPD-loop (Wang et al., 2020). Additionally, binding of an inhibitor may hinder the movement of the allosteric site, thereby impeding the conformational changes in the WPD-loop and leading to inhibition of PTP1B. These findings are consistent with our results. Moreover, in the α3 helix region, we observed a fluctuation of approximately 2.5 Å in protein without ligands, whereas this fluctuation reduced to about 1 Å on average in ligand-bound protein. Similarly, in the α6 helix region, we noted a fluctuation of about 1 Å without bound ligand compared to approximately 0.5 Å with bound ligand. These observations indicate that ligand binding enhances stability by reducing protein fluctuations.

**FIGURE 3:**
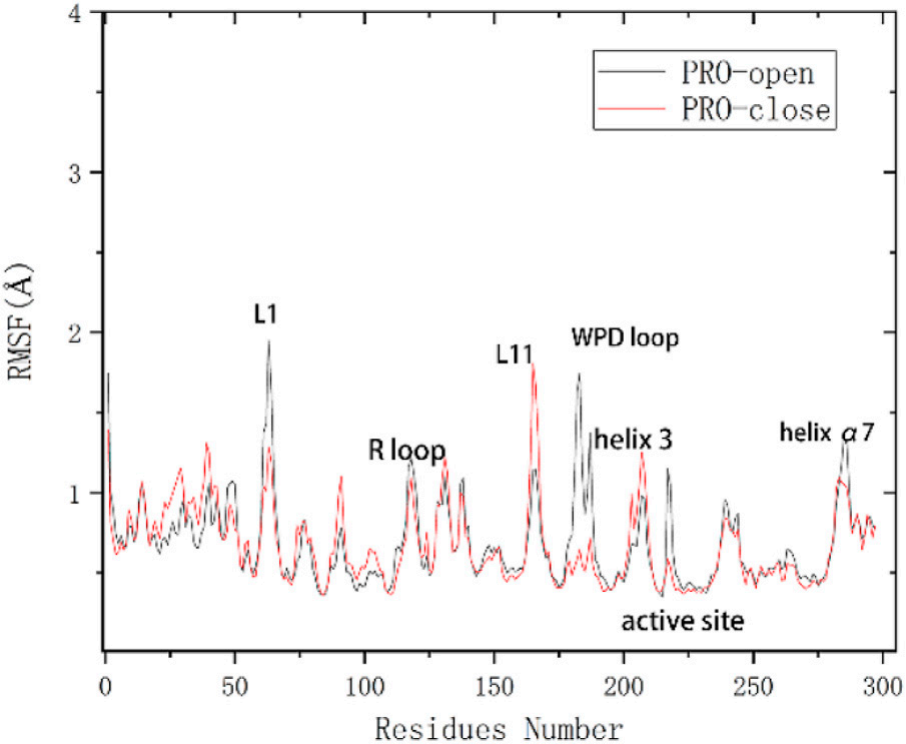
Flexibility changes in amino acid residues of the PTP1B protein (unbound inhibitor) (Wang et al., 2020).

Supplementary Figure S2 shows similar fluctuations between the different systems, which indicates that the complex systems are stable as a whole and do not lead to an increase in the structural motion of the residues. Under the restriction imposed by the inhibitor, the flexibility will naturally decrease if the inhibitor is successfully combined and interacts. It has been shown in previous studies (Wang et al., 2020) that the RMSF values of the WPD-loop of PTP1B are different between the PTP1B-open and close configuration. Open conformation has a RMSF value of about 2 Å. Close conformation has a RMSF value of about 0.5 Å (Figure 3). The results show that the RMSF value of the PTP1B protein (open conformation) in the WPD loop region of all complex systems after binding to the allosteric inhibitor is no higher than 1 Å. The results indicate that our docked ligand stably binds to the allosteric site of the PTP1B protein, affecting the movement of the helical region, which affects WPD-loop structural changes. The fluctuation of α3 helical residues in the complex-2 system is the smallest, and the fluctuation of α3 helical residues in the complex-1 system is the largest. This result show Ligand-2 exhibits the strongest intermolecular interaction, while Ligand-1 exhibits the weakest interaction. However, the RMSF curve for the complex-5 system at the α6 helix is small, showing that the intermolecular interactions of Ligand-2 and Ligand-3 are more potent than that of Ligand-1 and Ligand-5. Considering other compounds with poor inhibitory ability, substituents at C-23 and C-1 may affect the binding ability of the compounds (Figure 2).

Supplementary Figure S3 indicate that the RMSD values of the PTP1B complex system bound to the catalytic site. While Complex-4 exhibits larger fluctuations in the first 100 ns, it stabilizes in the subsequent 100 ns. The remaining compounds demonstrate general stability, with fluctuations ranging from 0.5–1.5 Å, leading us to conclude that overall system stabilization occurs in the latter 100 ns, during which subsequent binding free energy calculations were conducted. The RMSF values of the complex in Supplementary Figure S4, where the ligand binds to a competitive site, exhibit more pronounced overall fluctuations compared to those observed in the allosteric system. The final confirmation of the inhibitor’s type still necessitates validation through free energy analysis.

In addition, the other three compounds can also bind to the allosteric region of PTP1B to limit the movement of residues. However, whether their binding is stable and the energy effect is strong remains to be determined.

### 3.2 Energetic analysis of complex

#### 3.2.1 Binding free energy analysis

Subsequently, upon system stabilization, we computed the binding free energy of these triterpenoid compounds at their respective sites to assess and confirm their inhibitor type.

Table 8 shows the binding free energies (ΔG_bind_) for seven complexes were calculated by the MM-PB/SA approach. The ΔG_bind_ values of the seven compounds at the PTP1B allosteric site calculated are summarized in the Table 3 and the PTP1B catalytic site are summarized in Table 4. Table 3 shows that the order of binding energy is complex-1 > complex-2 > complex-5 > complex-3 > complex-7 > complex-6 > complex-4. ΔG_bind_ are −17.6 kcal mol^−1^, −17.3 kcal mol^−1^ −12.1 kcal mol^−1^, −9.4 kcal mol^−1^, −2.5 kcal mol^−1^, −2.2 kcal mol^−1^, 0.8 kcal mol^−1^, respectively, which are consistent with the trend of inhibition of PTP1B reported in the literature. However, the absolute values of the binding free energies in Table 4 are not consistent with the trends reported in the literature. Interestingly, in the course of our molecular dynamics simulations, we observed the some initial structures of ligands bound to the catalytic site, followed by their gradual transition away from the catalytic site towards the allosteric site over time. Based on the observed ligand movement during the MD process and the calculated binding free energy of the MM-PB/SA complex, it can be inferred that these compounds exhibit characteristics consistent with allosteric inhibition.

**TABLE 3:** Binding energy of seven complex at the PTP1B allosteric site (kcal mol^−1^).

	Complex-1	Complex-2	Complex-3	Complex-4	Complex-5	Complex-6	Complex-7
ΔE_ele_	−4.1	−6.5	−10.5	−6.3	−4.9	−9.5	−22.1
ΔE_vdw_	−47.3	−40.2	−36.2	−19.1	−37.9	−23.3	−32.6
ΔG_GB_	18.8	14.6	20.9	13.7	15.9	17.1	37.0
ΔG_SA_	−5.6	−4.7	−4.4	−2.4	−4.7	−2.8	−4.4
ΔG_pol_ ^a^	14.7	8.1	10.4	7.4	11.0	6.7	14.9
ΔG_nonp_ ^b^	−52.9	−44.9	−40.6	−21.5	−42.6	−26.1	−37.0
ΔG_MM-PB/SA_ ^c^	−38.2	−36.8	−30.1	−14.1	−31.3	−18.5	−22.2
–TΔS	20.6	19.5	20.7	14.9	19.2	16.3	19.7
G_bind_ ^d^	−17.6	−17.3	−9.4	0.8	−12.1	−2.2	−2.5

^a^ G_pol_ = E_ele_ + G_GB_.

^b^ G_nonp_ = E_vdw_ + G_SA_.

^c^ ΔG_MM-PB/SA_ = E_ele_ + G_GB_ + E_vdw_ + G_SA_.

^d^ ΔG_bind_ = ΔG_MM-PB/SA_ − TΔS.

**TABLE 4:** Binding energy of seven complexes at the PTP1B catalytic site (kcal mol^−1^).

	Complex-1	Complex-2	Complex-3	Complex-4	Complex-5	Complex-6	Complex-7
ΔE_ele_	−11.6	−1.7	−6.1	−9.6	−5.7	−2.0	−8.0
ΔE_vdw_	−31.7	−24.7	−24.5	−20.6	−25.7	−12.2	−23.7
ΔG_GB_	26.8	10.9	14.2	20.7	15.6	8.2	17.7
ΔG_SA_	−4.1	−3.0	−3.1	−2.7	−3.0	−1.5	−3.3
ΔG_pol_ ^a^	15.2	9.2	8.4	11.1	9.9	6.2	9.7
ΔG_nonp_ ^b^	−35.8	−27.7	−27.6	−23.3	−28.7	−13.7	−27.0
ΔG_MM-PB/SA_ ^c^	−20.7	−18.5	−19.5	−12.2	−18.7	−7.5	−17.4
–TΔS	19.4	18.2	16.3	16.6	16.8	12.5	16.7
G_bind_ ^d^	−1.3	−0.3	−3.2	4.4	−1.9	5.0	−0.6

^a^ G_pol_ = E_ele_ + G_GB_.

^b^ G_nonp_ = E_vdw_ + G_SA_.

^c^ ΔG_MM-PB/SA_ = E_ele_ + G_GB_ + E_vdw_ + G_SA_.

^d^ ΔG_bind_ = ΔG_MM-PB/SA_ − TΔS.

In these complexes, the binding free energy is mainly determined by ΔG_nonp_. ΔG_nonp_ is the sum of ΔE_vdw_ and ΔE_SA_, most of which comes from ΔE_vdw_. The value of ΔE_vdw_ gradually decreases with the difference and number of substituents of these ligands. ΔG_pol_ is the sum of ΔE_ele_ and ΔG_SA_, and entropy changes (-TΔS) adversely affect complexes and inhibitors. In other words, the larger the absolute value of G_bind_ is, the stronger the binding ability between the compound and the ligand indicates. Table 3 shows that the absolute value of ΔE_ele_ of complex-7 is greater than that of the other systems (−22.1 kcal mol^−1^), indicating that the contribution of electrostatic interaction in this system is relatively larger. However, complex-3 (−10.5 kcal mol^−1^) and complex-6 (−9.5 kcal mol^−1^) exhibit a medium degree of electrostatic interaction, and the ΔE_ele_ of the other systems is relatively smaller, this result indicates that the electrostatic interaction formed between amino acid residues and ligands within the system is relatively minor. However, relatively speaking, the absolute value of ΔE_vdw_ in all systems is extremely large. Possibly because the spatial position occupied by Ligand-1 is substantial, it can interact with more amino acids and has the highest van der Waals interaction. Whereas compounds with low activity demonstrate lower ΔE_ele_ and ΔE_vdw_ or higher ΔG_GB_.

Complex-1, complex-2, complex-3, and complex-5 have excellent binding free energies and IC_50_ values. These four compounds will be discussed in more detail later.

#### 3.2.2 Decomposition energy of different key residues

In order to explore residues that contribute significantly to receptor and ligand binding, we calculated the binding free energy for each residue in the four complexes (Ligand-1, Ligand-2, Ligand-3, Ligand-5) (Table 5). The energy of different key residues is divided into van der Waals energy, electrostatic interaction, polar solvation-free energy, and non-polar solvation-free energy (Supplementary Tables S1–S4). In this study, four compounds (Ligand-1, Ligand-2, Ligand-3, Ligand-5) have relatively stable free binding energies. Residues with binding free energy < -0.5 kcal mol^−1^ are considered vital residues.

**TABLE 5:** Binding free energies between PTP1B and seven potential molecules (kcal mol^−1^).

	Complex-1	Complex −2	Complex −3	Complex −5
ALA189	−0.872	−0.759	−1.016	−0.398
LEU192	−2.201	−2.397	−1.516	−2.108
ASN193	−1.270	−1.354	−1.325	−0.808
LEU195	−0.327	−0.689	−0.314	−0.308
PHE196	−2.065	−2.467	−2.167	−2.112
LYS197	0.192	0.154	−1.425	0.122
LEU232	−1.411	−0.649	0.093	−1.090
MET235	−0.993	−0.007	0.012	−0.356
ALA278	−0.615	−0.148	−0.197	−0.315
LYS279	−0.233	−0.268	−1.109	−0.187
PHE280	−2.380	−3.337	−1.968	−2.580
ILE281	−0.831	−1.550	0.003	−1.289

By analysing the contribution for each residue in the receptor, 12 residues (Ala189, Leu192, Asn193, Leu195, Phe196, Lys197, Leu232, Met235, Ala278, Lys279, Phe280, and Ile281) are essential. Besides van der Waals interaction rather than electrostatic contribute the most. In complex-1, Asn193 and Lys279 have higher electrostatic interactions. Met235 only showed higher binding capacity in complex-1, which may be attributed to the fact that Ligand 1 has a long side chain and is spatially closer to Met235. Lys197 and Lys279 have significantly lower free energies in complex-1, 2, and 5 with identical substituents at different positions than in complex-3. In addition, Phe196 and Phe280 are amino acid residues worthy of attention. Their free energy contribution in different systems is higher than that of other amino acid residues, especially in the Complex-2 system. This may indicate that two amino acids participate in the key residues for anchoring the compound.

### 3.3 Cluster analysis

**FIGURE 4:**
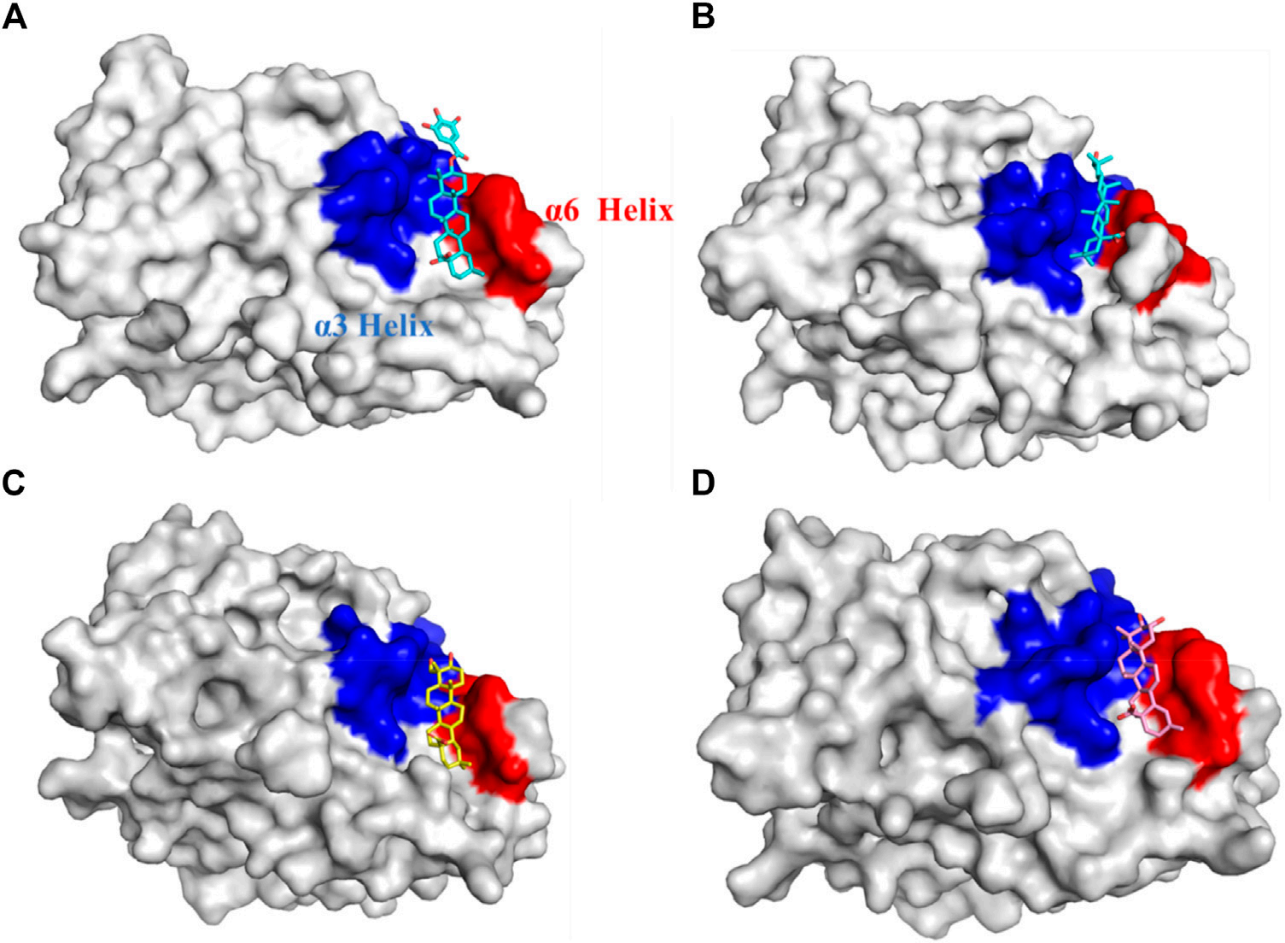
Cavity position and morphology of the four ligands in the PTP1B protein (Red: α6 Helix; Blue: α3 Helix). **(A)** Ligand-1 binds to the PTP1B protein. **(B)** Ligand-2 binds to the PTP1B protein. **(C)** Ligand-3 binds to the PTP1B protein. **(D)** Ligand-5 binds to the PTP1B protein.

Further exploration of the reasons for the strong binding ability and intermolecular interactions of these different inhibitors is required. It is possible to identify stable and representative conformations through cluster analysis in order to explore mechanisms of action between ligands and proteins. After the system is stabilized, the dominant conformation in each system is taken as the most representative conformation. Based on Table 6, cluster-1 is the dominant conformation. The Figure 4 clearly shows that the spatial differences between different ligands vary, while Ligand-1 can occupy the cavity position to a large extent, and can form more intermolecular interactions.

**TABLE 6:** Cluster analysis in four complexes systems.

No	Cluster1(%)	Cluster2(%)	Cluster3(%)	Cluster4(%)	Cluster5(%)
Complex-1	86.3	7.6	5.3	0.7	0
Complex-2	56.0	43.9	0.1	0	0
Complex-3	99.9	0.1	0	0	0
Complex-5	81.9	12.3	2.3	2.2	1.3

**FIGURE 5:**
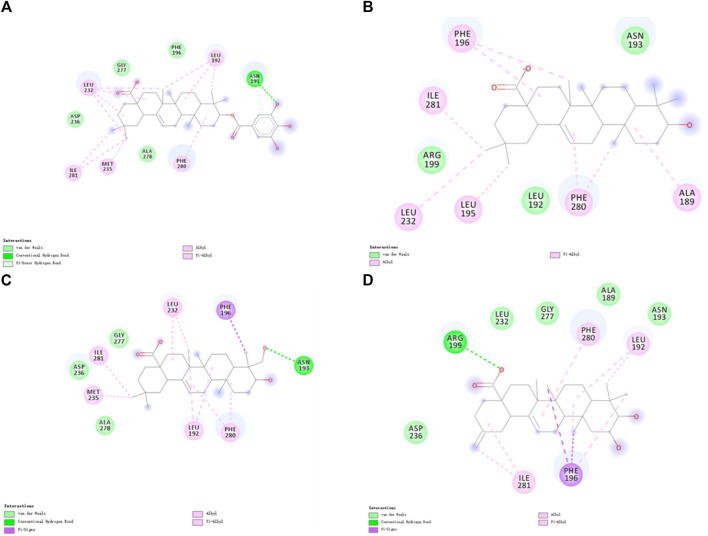
2D diagram of the interactions of four ligands with PTP1B proteins molecules. **(A)** 2D diagram of the interaction of Ligand-1 with PTP1B protein. **(B)** 2D diagram of the interaction of Ligand-2 with PTP1B protein. **(C)** 2D diagram of the interaction of Ligand-3 with PTP1B protein. **(D)** 2D diagram of the interaction of Ligand-5 with PTP1B protein.

**FIGURE 6:**
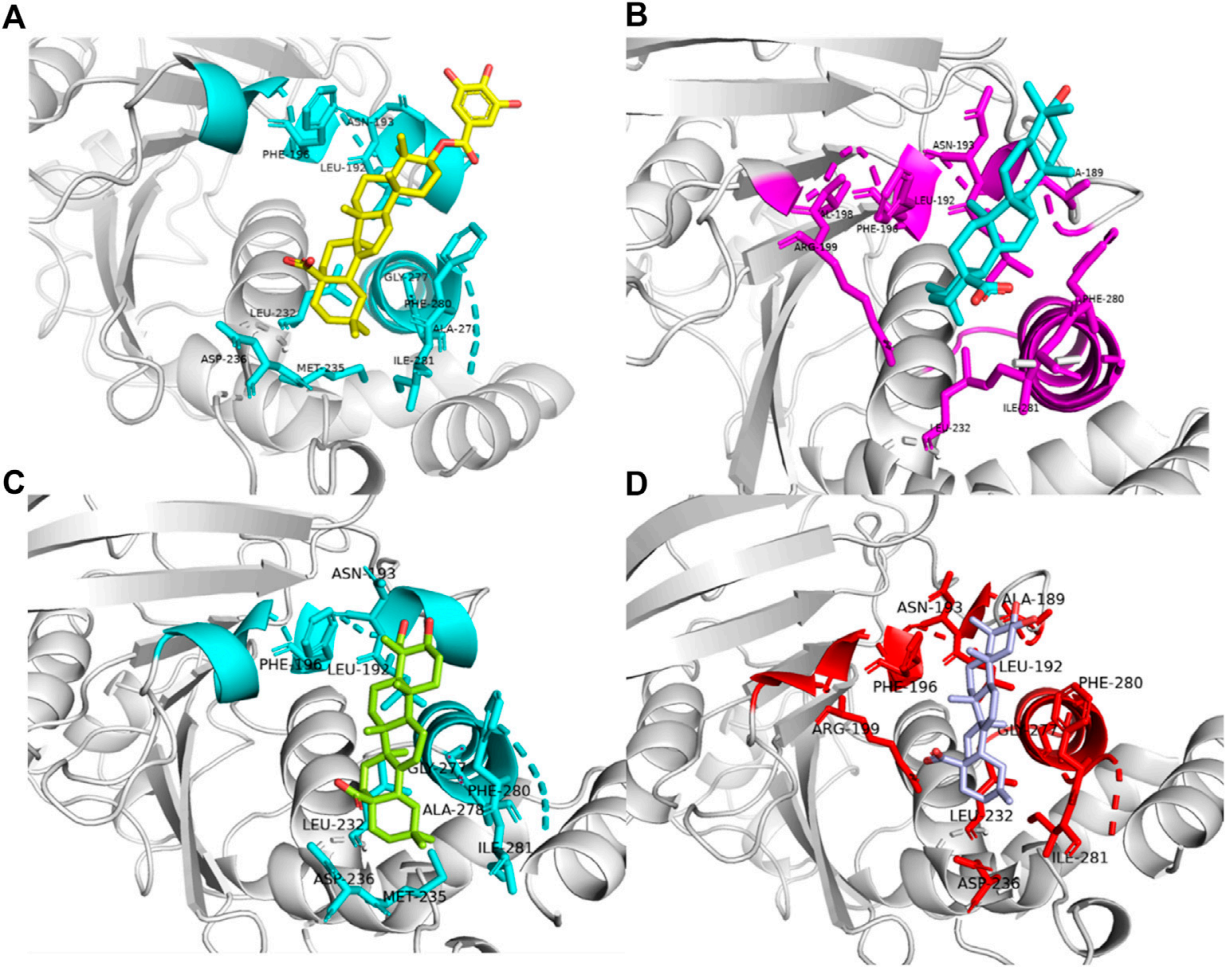
Binding patterns of four different ligands(Amino acid residues with interactive relationships are displayed in Stick mode, otherwise they are displayed in NewCartoon form, and different colors are used to distinguish Ligand-1, 2, 3 and 5.). **(A)** Ligand-1 binding to residues in complex-1 systems. **(B)** Ligand-2 binding to residues in complex-2 systems. **(C)** Ligand-3 binding to residues in complex-3 systems. **(D)** Ligand-5 binding to residues in complex-5 systems.

The detailed intermolecular interactions are shown in Figures 5, 6. A hydrophobic interaction is formed by the parent ring structure of these compounds (Leu192, Leu195, Phe196, Leu232, Met235, and Phe280), and some of the substituents or side chains can form hydrogen bonds (Asn193, Asp236, and Ala278). It is also important to note that different ligands form different interactions due to their parent rings and substitutes, and the properties of these substitutes may provide a basis for improving drugs. Despite the fact that Ligand-1 has fewer hydroxyl substituents than other ligands, it is still capable of exerting interaction with sufficient residues. Compared to Ligand-2, 3, and 5, it has limited substituents and side chains. However, the hydroxyl group at C-3 is a hydrophilic substitute that can stabilize the ligand’s existence, but Ligand-4 and Ligand-7 are deficient because C-2 and C-23 possess hydroxyl groups simultaneously. The methyl group of C-23 can exert a hydrophobic interaction on Leu192, thereby affecting the binding affinity. As a result, side chains can be added to C-23 to enhance the compound’s hydrophobic interaction.

Furthermore, by comprehensively considering the binding energy and structure, the simple structure-activity relationship reveals that the different positions of hydroxyl groups can impact the activity of the compounds, and the energy analysis demonstrates that there are indeed certain patterns. Following the augmentation in the quantity of hydroxyl groups, the polar desolvation energy of the inhibitor molecule is elevated, which is not beneficial for the contribution of the binding free energy. Ligand-1 possesses an O-Gal substituent, and the benzene ring establishes a π-Donor hydrogen bond with Asn193, thereby contributing a portion of the van der Waals interaction. Nevertheless, during the experiment, ligand-4 contains a preponderance of hydroxyl groups and deviate from the complex structure in the simulation process. Furthermore, given that the quantity of hydroxyl groups remains the same (Ligand-3, Ligand-5, Ligand-6), the compounds featuring hydroxyl groups at the C-23 or C-24 positions exhibit a greater ΔG_GB_. In the complex-3 compound system, the hydroxyl group at C-23 forms a conventional hydrogen bond with Asn193, thereby contributing a relatively strong binding free energy. The hydrophobic interaction furnished by Phe280 is relatively insignificant, whereas complexes 1, 2, and 5 suggest that Phe280 and Ile281 have a relatively high contribution to the binding free energy. Perhaps owing to the location of the hydroxyl groups, the ligand undergoes movement during the simulation process. This result implies that reducing the number of hydroxyl groups, or modifying the position of hydroxyl groups and enhancing the number of hydrophobic groups are all beneficial for increasing the stability of the compounds and ligands.

### 3.4 Combined mode of action analysis

**FIGURE 7:**
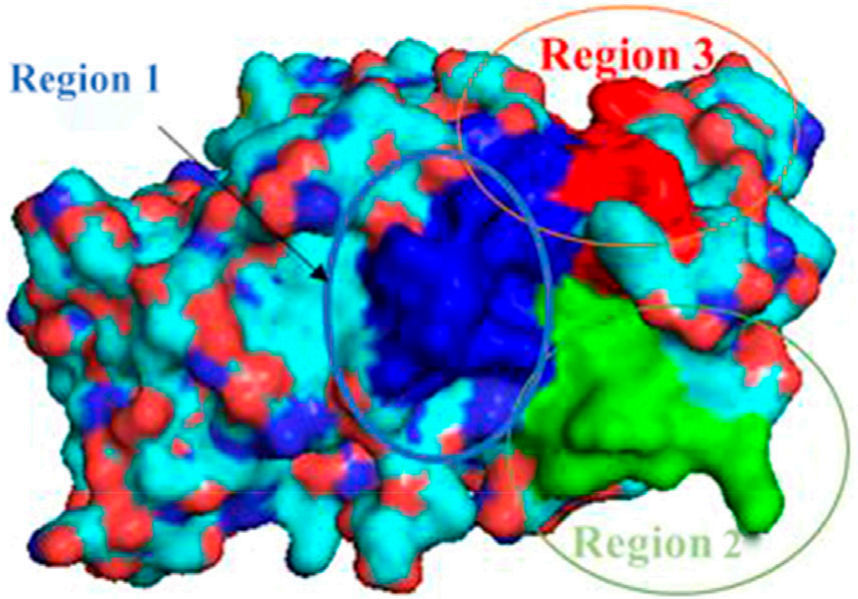
Region 1-3 of PTP1B protein.

For analysis and discussion, we divided the binding site into three helical regions to analyze the main interaction of these four natural product inhibitors in binding to PTP1B (Figure 7).

Region 1 is the α3 helix region (Glu186-Glu200). Cluster analysis results show that Leu192, Asn193, Phe196, Arg199 had interactions in region 1. During molecular docking, hydrogen bonds or van der Waals interactions were observed between Arg199 and the ligand. Arg199 is positively charged, and the ligand is negatively charged, forming a salt bridge interaction under electrostatic interaction. Molecular dynamics results show no corresponding salt bridge interaction formed in other complex systems. This may be because Ligand-1 has a long side chain. Different systems formed hydrophobic solid interactions with Phe196, indicating that pentacyclic triterpenoids are the core structure of anchoring inhibitors. Likewise, Leu192 forms hydrophobic interactions with different ligands and can interact with the parent structure of the ligand rather than its substituents or functional groups. We speculate that the Asn193 residue is structurally close to the inhibitor and only forms hydrogen bonds or van der Waals interactions. Compared with Complex-2 and Complex-3, Complex-1 exhibits this phenomenon most obviously. Asn193 forms a hydrogen bond with the O-Gal (galloyl group) at C-3 in Complex-1.

Due to the distance between region 2 (Gly220-Lys237) and the ligand, there is no obvious intermolecular interaction. Some amino acid residues still contribute to a higher binding free energy. Leu232 and Met235, for example, are between Complex-1, 2 and 5. The main interaction of these residues is hydrophobic, and we can consider increasing the length or substituents of the C chain structure at the head or tail of the parent structure. In order to form hydrogen bond interactions with Leu232 or Met235, act on the residue helix in region 2, or add substituents to the six-membered rings in the middle of the parent ring.

Region 3 consists of the α6 helical region (Ala264-Ile279). While the Phe280 substituent does not belong to this helical region, it produces a strong hydrophobic interaction in all systems. As phenylalanine itself has a benzene ring, the ligand compound moves under the influence of various residues, and finally, the six-membered ring and the benzene ring form a vertical stacking interaction. Phe280 contributes a high binding free energy through this interaction. As a result, we can consider modifying the six-membered ring, such as changing the single carbon-carbon bond to a double carbon-carbon bond to increase the intermolecular interaction.

Although Complex-4, 6, and 7 showed weak PTP1B inhibitory activity, we also explored their intermolecular interactions. The binding sites of these three compounds were identical, but they did not produce significant effects. Molecular dynamics results indicate that these three ligands only interact with a few amino acid residues. In particular, ligand-4 forms hydrophobic interactions with Asn193, Phe196, Lys197, Arg199, Glu200, Asp236, and Ile281. Different functional groups affect van der Waals and hydrogen bond interactions. The presence of hydroxyl groups at both C-1 and C-23 will have a significant impact on the reaction. Ligand-6 does not have this situation, but still has a low inhibitory activity. The framework consists of a terpene parent structure except for the hydrogen bond formed by the carboxyl substituent and Arg199. The structure of Ligand-6 is relatively simple, so although the compound forms an interaction with some key amino acids, the strength of the interaction is relatively low. Both Ligand-7 and Ligand-4 contain carboxyl groups at C-23 and C-1. The spatial positions of these two carboxyl groups are opposite, which may have a repulsive effect and prevent Ligand-7 from binding to the corresponding region. MD simulations verified that Ligand-4 and Ligand-7 deviated when interacting with the α3 helix region.

It may be possible to design inhibitors based on observations above. Increasing the interaction between Phe280 and Phe196, or considering the electrostatic interaction between Arg199 and Phe196, as well as considering the spatial position of the functional group, will facilitate the binding of the inhibitor to PTP1B.

### 3.5 Drug design and evaluation

#### 3.5.1 Growth or replacement of the molecular skeleton

LUDI algorithms can help new molecular skeletons or modify existing fragments to improve the binding capacity of ligands and PTP1B. We modified the design of fragments where small molecules can bind to critical regions of PTP1B. Finally, 16 compounds were obtained according to the fragment interaction screening of LUDI (Supplementary Figure S5).

#### 3.5.2 Rational design of inhibitors

In addition to the LUDI design, in order to obtain ligands with better affinity, we also carried out rational design of inhibitors. The rational design of inhibitors requires stable interactions with amino acid residues in key regions. In the design process, we divided into two steps (Singh et al., 2022): Substituents of drugs are modified, added, or removed to improve stability (Ma et al., 2021). The structure of the compound is optimized and treated to pass the blood-brain barrier. Display of cluster analysis results add some groups to hydrophilic substituents or transform some residues into hydrophobic groups to better interact with Phe280 and Phe196. Additionally, some large groups may be added to reduce P-glycoprotein (P-gp) efflux. By removing or modifying carboxyl groups, compounds can be more likely to cross the blood-brain barrier and form stronger intermolecular interactions with key residues.

Figures 5, 6 illustrate the position and molecular interaction of the cavity in which the ligand is located. Increasing the number of substituents for some untouched parts is also possible, which may increase the hydrophobic interaction. Consider adding some hydrophilic groups to the side exposed to the water environment to stabilize the ligand’s binding capacity. In addition, there are some other charged groups in the helix design, and the extension of the carboxyl group of the original compound and transformation into an electrostatic interaction will also contribute to increasing binding free energy. In addition, to enhance the binding ability of the ligand, substituents or extended side chains are added to the side close to the helix, which allows the ligand to fill the protein cavity, resulting in a higher number and stability of interactions with the α6 helix residues (Glu276, Gly277, Ala278, Ile281).

Furthermore, as per previous research reports, the -CF_3_ groups have been shown to enhance ligand binding affinity through orthogonal dipolar C-F···C = O interactions with the protein’s backbone carbonyl groups. This effect has also been demonstrated in a study by Ledy De-la-Cruz-Martinez et al. (De-la-Cruz-Martínez et al., 2021), where compounds containing -CF_3_ groups significantly boosted PTP1B inhibitory activity. Additionally, the -CF_3_ groups can increase lipophilicity and improve blood-brain barrier permeability, aligning with our anticipated outcomes (Design-11 and Design-12). We can validate this through computation of binding free energy.

#### 3.5.3 ADMET prediction

**FIGURE 8:**
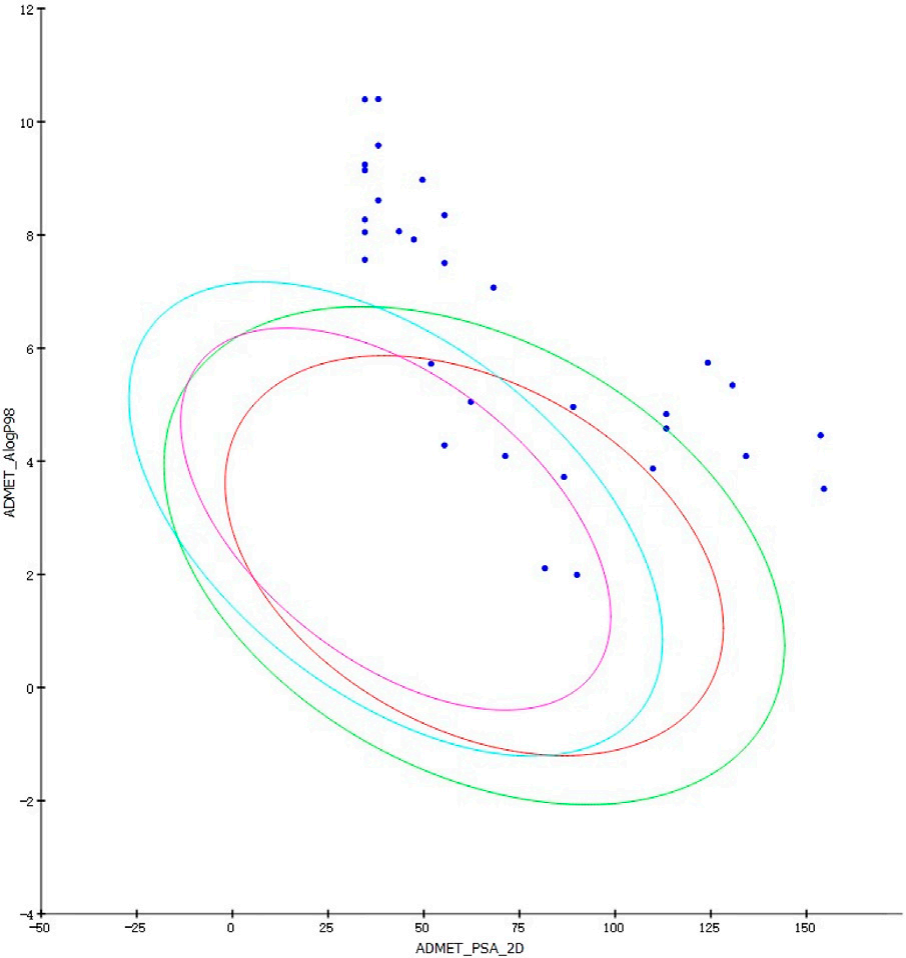
ADMET predictions for LUDI and rationally designed drugs. Blue ellipse: 99% confidence interval of the BBB model. Green ellipse: 99% confidence interval of the HIA model.

By modifying the ligands and substituents’ structure, we obtained 16 LUDI-modified compounds and 14 rationally designed compounds and predicted their ADMET properties. Figure 8 shows that only a small number of design results fall within the 99% confidence interval of the BBB, HIA, and Log(Sw) model, and this molecule’s prediction is considered relatively reliable. Table 7 shows the prediction details of ADMET for selected compounds. LUDI designed a total of 16 compounds, rationally designed 14 compounds, and the compounds that can pass ADMET may have the possibility of successful design.

**TABLE 7:** ADMET properties of 7 compounds.

Name	ADMET_Absorption_Level^a^	ADMET_Solubility^b^	ADMET_Solubility _level	ADMET_BBB^c^	ADMET_BBB_Level^c^
LUDI-6	0	2	−5.967	0.293	1
LUDI-7	0	1	−7.423	0.793	0
Design-5	0	2	−4.418	−0.962	3
Design-11	0	1	−6.632	0.421	1
Design-12	0	2	−5.422	−0.375	2
Design-13	0	2	−4.515	−0.794	3
Design-14	0	2	−4.893	−0.018	2

^a^ Level:0 ADMET_Absorption_T2_2D < 6.1261 (inside 95%) (Good absorption).

^b^ Level:1, −8.0 < log(SW)<-6.0, very low, but possible. Level:2, −6.0 < log(SW)<-4.1, low.

^c^ Level:0, Brain-Blood ratio greater than 5:1, very high. Level:1, Brain-Blood ratio between 1:1 and 5:1, high. Level:2, Brain-Blood ratio between 0.3:1 and 1:1, medium.

**FIGURE 9:**
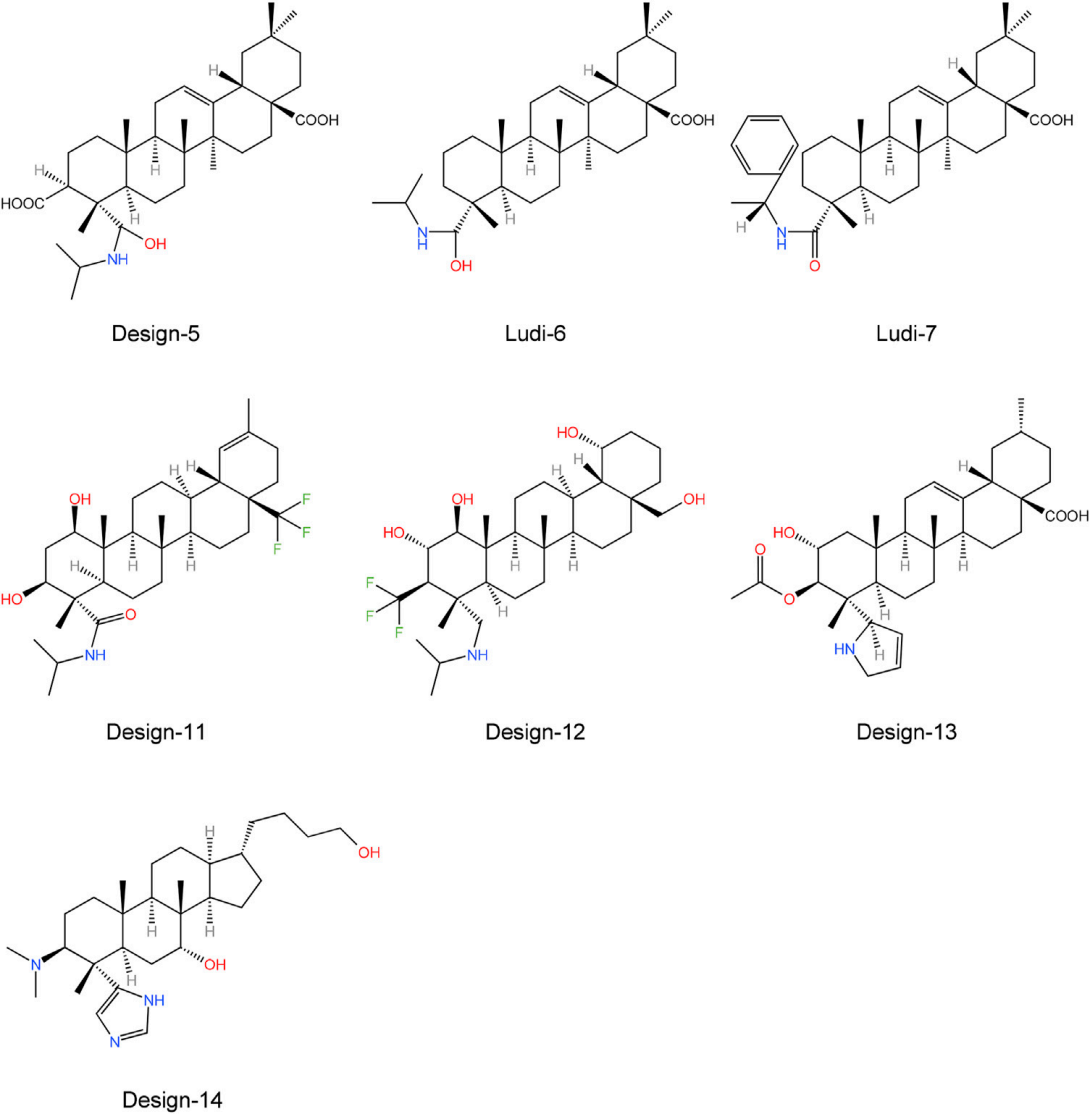
Molecular structure of seven potential PTP1B inhibitors.

Finally, we obtained seven compounds (LUDI-6, LUDI-7, Design-5, Design-11, Design-12, Design-13, and Design-14) (Figure 9) and docked them to the PTP1B protein, then performed a 200 ns MD simulation and calculated the binding free energy. The structures of other compounds are shown in Supplementary Figures S11, S12.

#### 3.5.4 Molecular dynamics simulations of designed compounds

An MD simulation was performed on the 7 designed compounds and the MD conditions were identical to those used for the 7 natural inhibitors of PTP1B.Supplementary Figures S5, S6 show the RMSD values of the designed drug system, and the fluctuation range of these seven designed drugs is between 0.75 and 1.75 Å, fluctuating about 1 Å. Supplementary Figures S7, S8 show that the RMSF value of the designed drug system has decreased fluctuations in the α3 and α6 helix regions, and it can be speculated that these compounds can be stably bound in the α3 and α6 helix regions. After conducting molecular dynamics simulations, the designed compounds were compared with the original protein structure (Supplementary Figures S9, S10). The results indicated that only the Design-12 complex exhibited higher fluctuations than the protein without the ligand at Thr230-Leu250, while the other complex systems displayed a more stable trend. This may be attributed to the force-inducing effect of the -CF_3_ group (De-la-Cruz-Martínez et al., 2021).

#### 3.5.5 Energy calculation of inhibitors

Table 8 shows the free binding energies of these seven potential inhibitor ligands. It can be seen that the ΔG_bind_ of LUDI-7 and Design-14 is poor. Due to their weaker binding free energies than complex-1 and complex-2, LUDI-6 and Design-5 were also excluded from the design (Figure 9).

**TABLE 8:** Free energy for binding of PTP1B protein to 7 potential inhibitor ligands.

	LUDI-6	LUDI-7	Design-5	Design-11	Design-12	Design-13	Design-14
ΔE_ele_	15.0	13.6	20.6	−13.8	−15.4	−15.5	−14.7
ΔE_vdw_	−45.3	−44.1	−42.3	−44.2	−44.5	−42.5	−28.4
ΔG_GB_	−4.6	2.1	−9.2	19.7	23.9	21.6	22.7
ΔG_SA_	−5.3	−5.3	−5.2	−5.2	−5.2	−4.9	−3.7
ΔG_pol_ ^a^	9.7	15.7	11.4	5.9	8.5	6.1	8.0
ΔG_nonp_ ^b^	−50.6	−49.4	−47.5	−49.4	−49.7	−47.4	−32.1
ΔG_MM-PB/SA_ ^c^	−37.2	−33.7	−36.1	−43.5	−41.3	−41.4	−24.1
–TΔS	23.2	24.3	21.8	19.9	22.2	20.4	20.4
G_bind_ ^d^	−14.0	−9.44	−14.3	−23.6	−19.1	−21.0	−3.7

^a^ G_pol_ = E_ele_ + G_GB_.

^b^ G_nonp_ = E_vdw_ + G_SA_.

^c^ ΔG_MM-PB/SA_ = E_ele_ + G_GB_ + E_vdw_ + G_SA_.

^d^ ΔG_bind_ = ΔG_MM-PB/SA_ − TS.

MM-PB/SA calculations were performed for these compounds by selecting the last 100 ns stable trajectory. The three compounds with the best free binding energy are Design-11, 12, and 13, which are −23.6 kcal mol^−1^, −19.1 kcal mol^−1^, and −21.0 kcal mol^-1^, respectively. A comparison of the decomposition energies of key residues in different complex systems is shown in Supplementary Tables S5–S11. The contributions of the three inhibitors to the van der Waals interaction and electrostatic interaction were significantly greater than those of the previous compounds. Intermolecular interactions and energies indicate that the design of these three compounds merits further investigation. We believe that the results of these designs will provide a potential idea and a more convenient theoretical basis for the design of future drugs. Our findings, derived from the integration of free energy calculations, align with those reported by Ledy De-la-Cruz-Martinez et al., providing further evidence that the -CF_3_ group can enhance the efficacy of PTP1B inhibitors (De-la-Cruz-Martínez et al., 2021).

### 3.6 TOPKAT and druggability analysis

To evaluate the compounds’ toxicity, use the TOPKAT module in DS2020 and utilize the Drugflow platform to analyze druggability. Artificial intelligence (AI) is utilized on the Drugflow platform, which includes machine learning algorithms and deep learning algorithms that are more accurate, resulting in more accurate predictions. Using Drugflow’s selected MERT (Pre-train) method, we will analyze the druggability of these LUDI-designed and rationally designed compounds (Table 9).

**TABLE 9:** Druggability analysis and toxicity test results of designed drugs.

Name	Lipinski rule^a^	PPB^b^	BBB^c^	H-HT^d^
LUDI-6	Reject	0.938	0.078	0.001
LUDI-7	Reject	0.97	0.086	0.278
Design-5	Reject	0.93	0	0
Design-11	Reject	0.945	0	0.711
Design-12	Accepted	0.842	0.001	0.006
Design-13	Accepted	0.853	0.337	0.161
Design-14	Accepted	0.758	0.001	0.999

Name	LogS^e^	LogP^f^	LogD^g^	SAscore^h^	F(20%)^i^	Fu^j^
LUDI-6	−4.598	4.962	3.782	4.721	0.765	0.019
LUDI-7	−5.44	5.469	4.43	5.218	0.99	0.018
Design-5	−4.266	4.031	3.214	5.195	0.09	0.016
Design-11	−4.573	4.853	4.186	5.438	1	0.018
Design-12	−4.582	3.653	2.875	5.485	0.99	0.01
Design-13	−3.968	3.234	2.412	5.966	0.616	0.009
Design-14	−3.324	3.789	2.941	5.082	0.055	0.008

Name	CYP1A2-inh^k^	CYP2C9-inh	CYP2C19-inh	CYP2D6-inh	CYP3A4-inh	DILI^l^	LD50(g kg-1)^m^
LUDI-6	0	0	0	0	0	0	2.680
LUDI-7	0	0	0.01	0	0.017	0	4.504
Design-5	0	0	0	0	0	0	3.569
Design-11	0	0	0	0	0.85	0.002	≥10
Design-12	0	0	0	0	0	0	≥10
Design-13	0.001	0.032	0	0.272	0	0	0.3071
Design-14	0.054	0.049	0.196	0.998	0.389	0.868	8.699

^a^ MW (molecular mass including hydrogen atoms) ≤500; LogP (logarithm of octanol/water partition coefficient) ≤5; Hacc (hydrogen bond acceptor) ≤10; Hdon ≤ 5 (hydrogen bond donor) is considered Acceptable, otherwise it is rejected.

^b^ Plasma Protein Binding; If the PPB, value is higher than 90%, the therapeutic index may be lower. (0–1).

^c^ blood brain barrier; The higher the value, the more likely it is that it cannot penetrate the blood-brain barrier. (0–1).

^d^ Human Hepatotoxicity; The higher the number, the more likely hepatotoxicity is present. (0–1).

^e^ The logarithm of the solubility of an aqueous solution. The optimal value is -4-0.5 log mol/L.

^f^ octanol/water partition; The optimal value is 0–3.

^g^ LogP value at physiological pH 7.4, optimal is 1–3

^h^ Synthetic accessibility score; SAscor ≥ 6:Difficult to synthesize, SAscore < 6:Easy to synthesize.

^i^ bioavailability (20%); category 1: F20%+,bioavailability < 20%; category 0: F20%-, bioavailability > 20%.The probability that the output value is F20+.

^j^ Unbound fraction of plasma: low 0.05, medium 0.05–0.2, high > 0.2.

^k^ Cytochrome P450 inhibitor, category 1:inhibitor, category 0: non-inhibitor. The output value is the probability of the inhibitor.

^l^ Drug induced liver injury; category 1: ILI, high-risk drugs, category 0: DILI, low-risk drugs. The output value is the probability of poisoning.

^m^ Median Lethal Dose. LD50 > 2000 mg/kg, non-toxic; LD50 < 50 mg/kg, very poisonous (Alberga et al., 2019).

Through toxicity testing and druggability analysis of the compounds, we finally found that compound Design-12 almost met all our needs, not only compared to Ligand-1 (−17.6 kcal mol^−1^) with a higher IC50 (2.10 μM) has better binding ability (Design-12, -19.1 kcal mol^−1^). Although the binding ability of Design-13 is less than that of Design-11 and Design-13, it has a very high safety risk, whether it is drug loss or liver damage. The binding free energy of Design-11 is the best of all compounds in terms of toxicity and oral LD50 in rats. The compound also exhibits selectivity for PTP1B, liver toxicity and oral LD50 in rats. Lipinski Rule does not accept it because it is relatively excellent.

Furthermore, the drugs designed in the experiment are easy to synthesize. Consequently, our research indicates that Design-12 may be an effective treatment for type 2 diabetes. The Design-11 has poor efficacy, but it still has the potential to be useful. In addition, Design-13 has excellent inhibitory properties.

## 4 Discussion

In this study, initially, natural pentacyclic triterpene PTP1B inhibitors sharing the identical parent molecule were retrieved from previous literature reports (Xu et al., 2018; Huang et al., 2022), and their interaction sites and interaction patterns were investigated through molecular docking and molecular dynamics. Subsequently, the binding free energy and the total interaction patterns were analyzed in accordance with the notions and approaches of Wang and Zheng (2018). Nevertheless, the research they carried out has constraints. For example, they investigated the inhibitory impacts of multiple compounds on the protein; however, whether the same holds true for compounds of the same type remains to be deliberated. Additionally, only straightforward toxicity verification was performed. This study aims to explore the binding mode of a class of compounds and conduct structural analysis by comparing compounds with the same parent molecule but different substituents, a method that is largely applicable to other pentacyclic triterpenoid compounds as well. Furthermore, the structural analysis of the system can investigate the motion-bound mode of the allosteric site of PTP1B. For instance, Phe196 establishes π-π stacking interactions and hydrophobic interactions with the pentacyclic triterpene, which can be detected in all the simulated systems. This phenomenon also suggests that the framework of the triterpenoid compound functions in anchoring the compound. Additionally, Phe280 can also establish supplementary π-stacking interactions in certain systems. This is similar to the results obtained by De-la-Cruz-Martínez et al. (2021); Ali et al. (2023); Sánchez-Alonso et al. (2021). Unfortunately, their study simply failed to account for the fact that the spatial positions of substituents also exert an influence on the activity of compounds, such as the matter regarding the hydrophilicity or hydrophobicity of the substituents in the directions at the C-3 and C-4 regions. Nevertheless, the structural analysis in this research might be capable of elucidating this aspect and conducting a more meticulous analysis of the spatial positions and states of the compounds. After conducting structural analysis, based on its conclusions, it is feasible to investigate whether elongating the length of substituents can enhance the stability of the compound or make modifications that are more liable to interact with the protein, thereby offering new concepts and insights for the compound to bind more readily to the protein. The results also suggest that the compounds subsequent to structural analysis frequently exhibit more superior binding capabilities. As hypothesized earlier, reducing the number of hydroxyl groups truly can enhance the contribution of binding free energy and attain a more stable system. The Design-12 compound did not decrease the number of hydroxyl groups but rather altered the position of the hydroxyl groups, and the contribution of the binding free energy was higher. The results manifested that the position of the hydroxyl groups could indeed cause the compound to undergo deflection motions in distinct directions.

## 5 Conclusion

In this study, Molecular dynamics (MD) simulation and Molecular Mechanics Poisson Boltzmann Surface Area (MM-PB/SA) were used to study the binding patterns of allosteric sites between compounds and PTP1B previously reported. By comparing the configurations of different ligands and the corresponding IC_50_ values, we found that the van der Waals interaction contributes the most energy, while electrostatic interactions contribute less energy. If the inhibitor can interact well with the critical residues of the corresponding site, it can show high inhibitory ability. Pentacyclic triterpenoids form mostly hydrophobic interactions with the α3 and α6 helical regions of PTP1B, among which Phe196 and Phe280 contribute the most hydrophobic interactions. We design 30 novel inhibitor molecules through LUDI and rational design methods. We calculated the binding free energy and analyzed the drugability of the designed LUDI-6, LUDI-7, Design-5, Design-11, Design-12, Design-13 and Design-14. The results showed that Design-12 was the most optimal inhibitor. Although Design-14 has good druggability analysis results, its binding free energy is deficient, thus, it will not be considered. There is a more stable binding energy in Design-12, which shows a reasonable range in various druggability analysis indicators. Even though the results of Design-11 are not acceptable, it has a higher binding free energy than Design-12, indicating a more significant inhibitory effect. The Design-13 also has excellent properties, but its oral LD50 in rats is inferior to that of Design-12. Our findings will provide new understanding and suggestions for future researchers to design new and effective inhibitors of PTP1B.

## Data Availability

The original contributions presented in the study are included in the article/Supplementary Material, further inquiries can be directed to the corresponding authors.
