# Supplementary material for: Theoretical study on the design of allosteric inhibitors of diabetes associated protein PTP1B
Source: Front Pharmacol. 2024 Aug 22;15:1423029. doi: 10.3389/fphar.2024.1423029 (PMC11374740; doi:10.3389/fphar.2024.1423029)
Supplement: Supplementary file 1 [file DataSheet1.docx]

**Analysis of small molecule ligand systems and design of compounds Zhen-yang Liu, Hong-wei Gao*, Jiu-yu Zhan***

*School of Life Science, Ludong University, Yantai, Shandong, 264025, China.*

**Fig.S1 RMSD values (Å) for binding seven ligands to the PTP1B protein in a 200ns simulation.**

**Fig.S2** **RMSF values (Å) for binding seven ligands to the PTP1B protein in a 200ns simulation.**

**
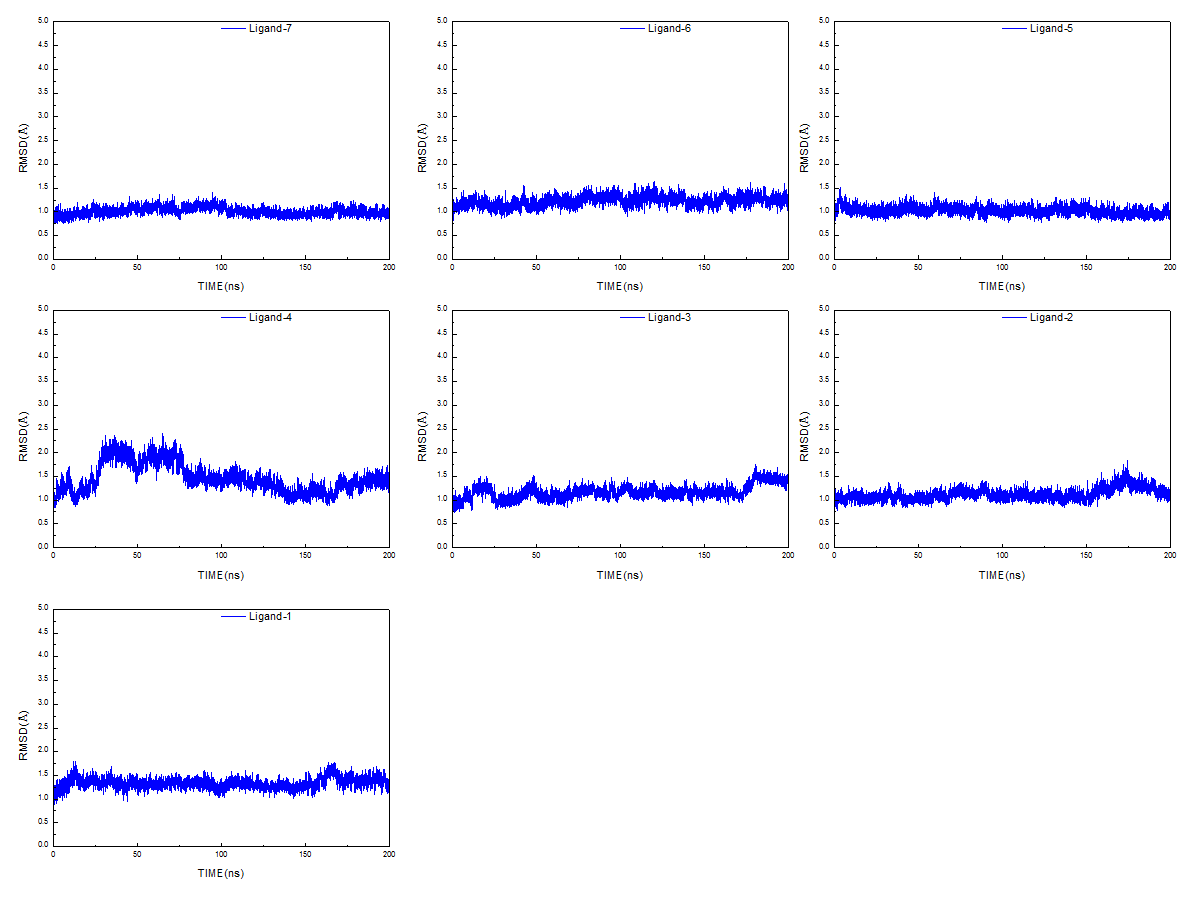
****Fig.S3 RMSD values (Å) for binding seven ligands to the PTP1B protein in a 200ns simulation(catalytic site).**


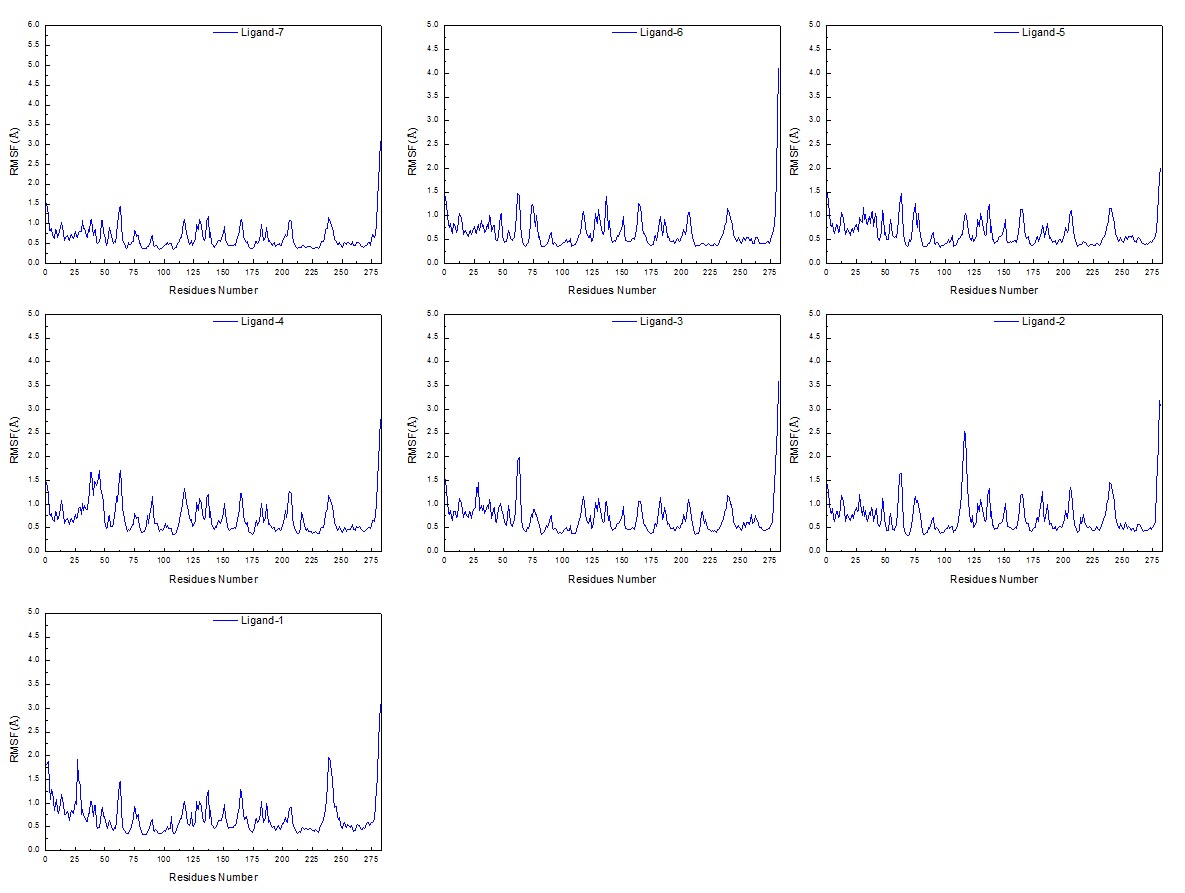


**Fig.S4** **RMSF values (Å) for binding seven ligands to the PTP1B protein in a 200ns simulation (catalytic site).**


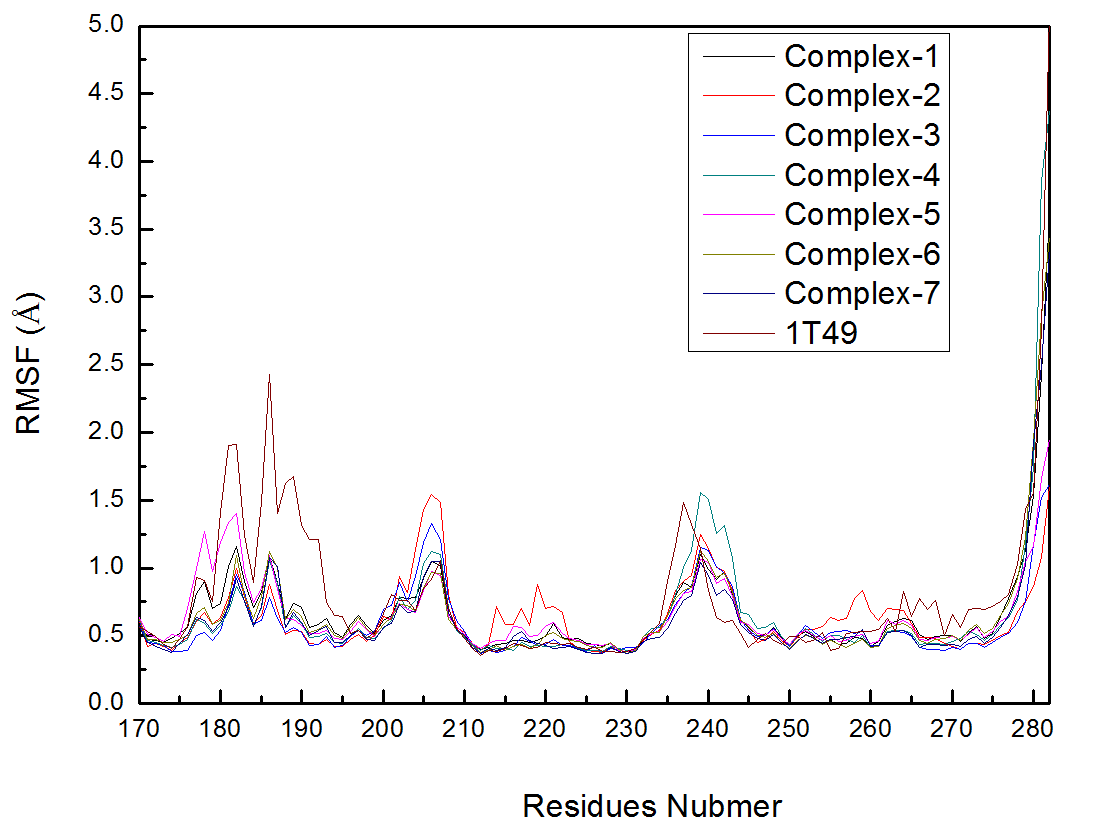


**Fig.S5 The comparison chart of RMSF for diverse systems and unbound ligand proteins.**

**Fig.S5 RMSD values (Å) for LUDI-6 and LUDI-7 systems during 200ns molecular dynamics simulation**

**Fig.S6** **RMSD values (Å) for the designed drug systems during 200ns molecular dynamics simulation**

**Fig. S7 RMSF value (Å) at each residue position for LUDI-6 and LUDI-7.**

 **Fig. S8 RMSF value (Å) at each residue position for the design drug.**
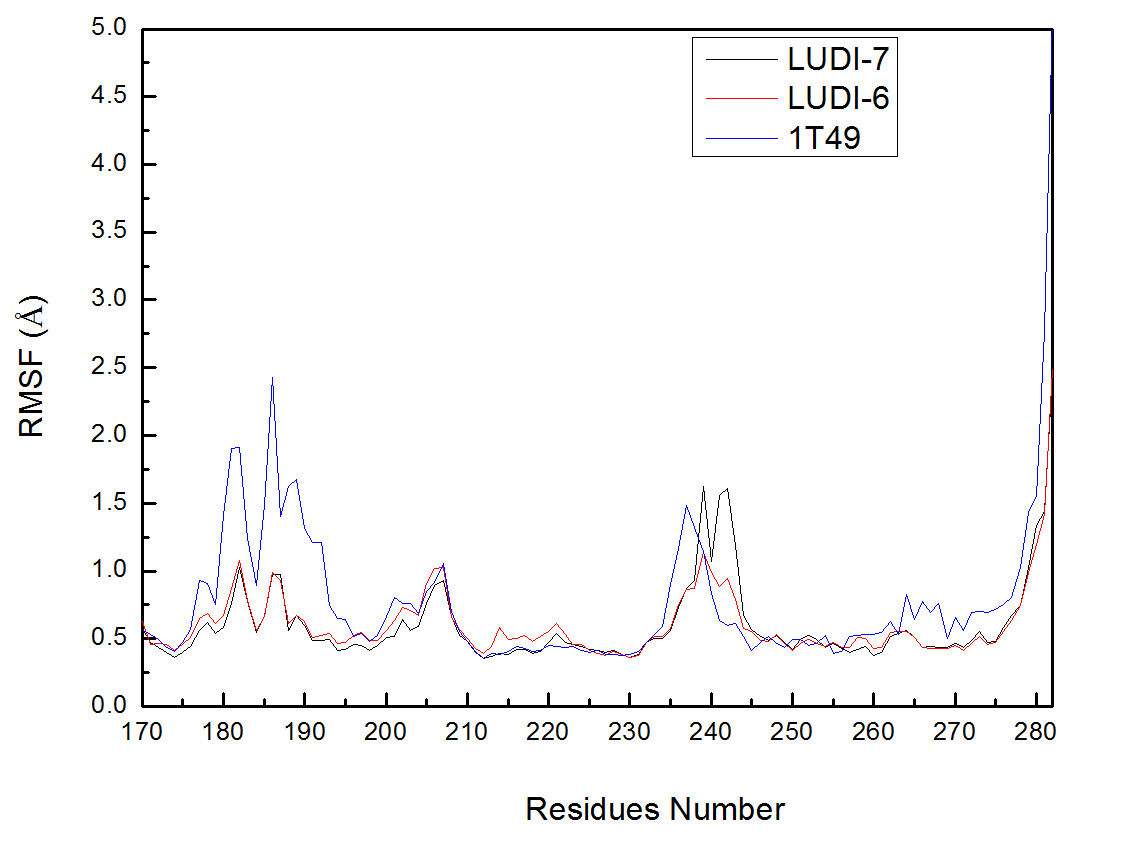


**Fig. S9** **The comparison chart of RMSF for the LUDI-6 and LUDI-7 systems and unbound ligand proteins**


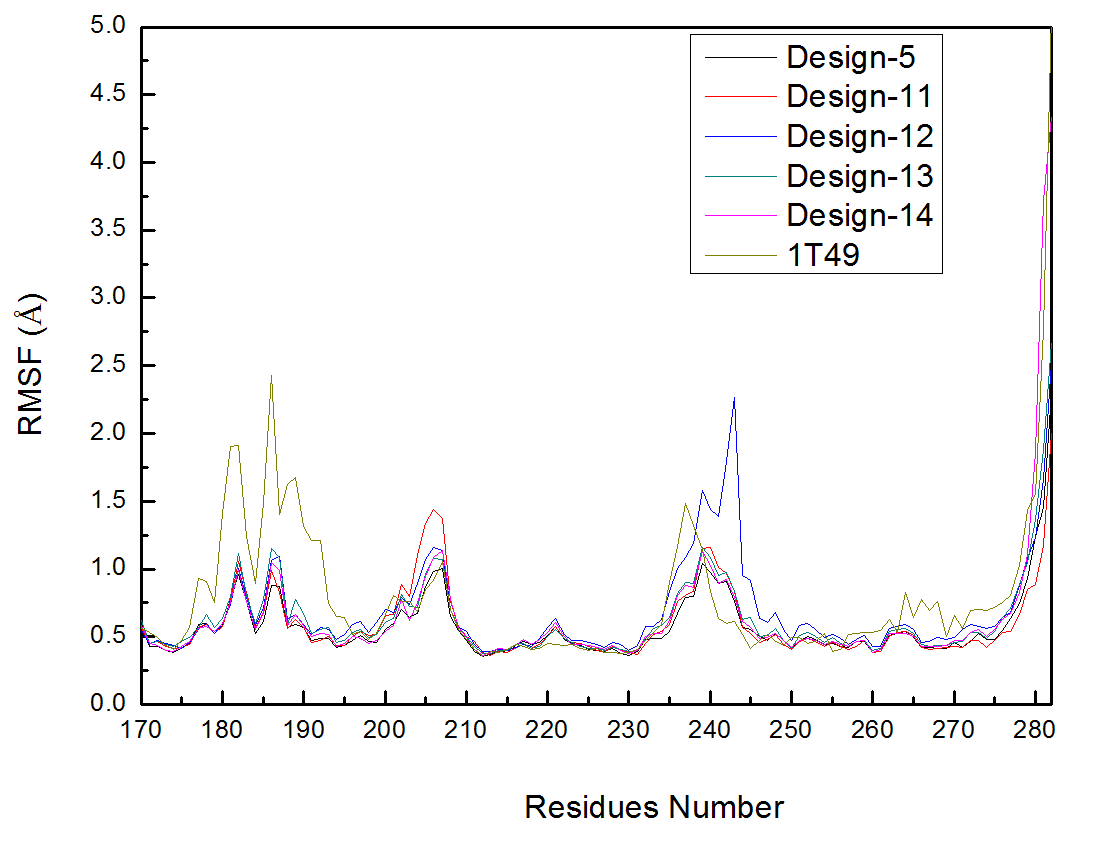


**Fig. S10** **The comparison chart of RMSF for the rationally designed compound systems and unbound ligand proteins**

**Fig.S11 Structures of new molecules obtained from LUDI modification.**

**Fig.S12 Structures of new molecules obtained from rational design.**

**Table.S1 Decomposition energy of key residues of complex-1**

|  | Vdw | Ele | Pol | Non-pol | Total |
| --- | --- | --- | --- | --- | --- |
| ALA189 | -0.747 | -0.022 | 0.036 | -0.140 | -0.872 |
| LEU192 | -2.251 | -0.201 | 0.468 | -0.217 | -2.201 |
| ASN193 | -1.634 | -0.815 | 1.454 | -0.275 | -1.270 |
| LEU195 | -0.290 | 0.006 | -0.035 | -0.008 | -0.327 |
| PHE196 | -2.564 | -0.084 | 0.982 | -0.399 | -2.065 |
| LYS197 | -0.134 | -0.021 | 0.349 | -0.002 | 0.192 |
| LEU232 | -2.164 | -0.164 | 1.123 | -0.205 | -1.411 |
| MET235 | -1.371 | 0.040 | 0.453 | -0.116 | -0.993 |
| ALA278 | -0.655 | -0.034 | 0.111 | -0.037 | -0.615 |
| LYS279 | -0.322 | -1.175 | 1.274 | -0.010 | -0.233 |
| PHE280 | -3.259 | -0.252 | 1.639 | -0.509 | -2.380 |
| ILE281 | -1.030 | 0.042 | 0.296 | -0.139 | 0.831 |

**Table.S2 Decomposition energy of key residues of complex-2**

|  | Vdw | Ele | Pol | Non-pol | Total |
| --- | --- | --- | --- | --- | --- |
| ALA189 | -1.139 | -0.123 | -0.684 | -0.180 | -0.759 |
| LEU192 | -2.653 | -0.162 | 0.593 | -0.175 | -2.397 |
| ASN193 | -2.693 | -0.297 | 2.006 | -0.371 | -1.354 |
| LEU195 | -0.742 | 0.058 | 0.024 | -0.028 | -0.689 |
| PHE196 | -2.789 | -0.040 | 0.024 | -0.028 | -0.689 |
| LYS197 | -0.143 | 0.347 | -0.049 | -0.001 | 0.154 |
| LEU232 | -0.705 | 0.045 | 0.075 | -0.064 | -0.649 |
| MET235 | -0.031 | 0.025 | -0.001 | -0.000 | -0.007 |
| ALA278 | -0.147 | 0.034 | -0.034 | -0.000 | -0.148 |
| LYS279 | -0.122 | -0.235 | 0.090 | -0.000 | -0.268 |
| PHE280 | -3.255 | -0.543 | 0.878 | -0.416 | -3.337 |
| ILE281 | -1.642 | 0.471 | -0.171 | -0.208 | -1.550 |

**Table.S3 Decomposition energy of key residues of complex-3**

|  | Vdw | Ele | Pol | Non-pol | Total |
| --- | --- | --- | --- | --- | --- |
| ALA189 | -1.108 | -0.037 | 0.339 | -0.210 | -1.016 |
| LEU192 | -1.704 | 0.144 | 0.137 | -0.093 | -1.516 |
| ASN193 | -2.851 | 0.281 | 1.601 | -0.355 | -1.325 |
| LEU195 | -0.160 | 0.164 | -0.318 | -0.000 | -0.314 |
| PHE196 | -2.308 | 0.210 | 0.143 | -0.212 | -2.167 |
| LYS197 | -1.057 | -2.893 | 2.674 | -0.149 | -1.425 |
| LEU232 | -0.058 | 0.029 | -0.063 | -0.000 | -0.093 |
| MET235 | -0.010 | 0.010 | 0.013 | 0.000 | 0.012 |
| ALA278 | -0.126 | 0.057 | -0.129 | 0.000 | -0.197 |
| JLYS279 | -1.225 | 1.133 | -0.833 | -0.184 | -1.109 |
| PHE280 | -2.723 | 0.075 | 1.085 | -0.404 | -1.968 |
| ILE281 | -0.084 | 0.016 | 0.071 | -0.000 | 0.003 |

**Table.S4 Decomposition energy of key residues of complex-5**

|  | Vdw | Ele | Pol | Non-pol | Total |
| --- | --- | --- | --- | --- | --- |
| ALA189 | -0.420 | -0.058 | 0.149 | -0.069 | -0.398 |
| LEU192 | -2.195 | -0.021 | 0.329 | -0.220 | -2.108 |
| ASN193 | -1.390 | -0.186 | 0.953 | -0.186 | -0.808 |
| LEU195 | -0.248 | 0.068 | -0.123 | -0.005 | -0.308 |
| PHE196 | -2.623 | 0.007 | 0.917 | -0.412 | -2.112 |
| LYS197 | -0.110 | -0.059 | 0.291 | -0.000 | 0.122 |
| LEU232 | -1.518 | -0.104 | 0.689 | -0.158 | -1.090 |
| MET235 | -0.467 | 0.063 | 0.082 | -0.033 | -0.356 |
| ALA278 | -0.264 | 0.006 | -0.055 | -0.002 | -0.315 |
| LYS279 | -0.255 | 0.179 | -0.108 | -0.003 | -0.187 |
| PHE280 | -3.284 | -0.207 | 1.465 | -0.554 | -2.580 |
| ILE281 | -1.264 | -0.077 | 0.238 | -0.186 | -1.289 |

**Table.S5 Decomposition energy of key residues of Ludi-6**

|  | Vdw | Ele | Pol | Non-pol | Total |
| --- | --- | --- | --- | --- | --- |
| ALA189 | -0.526 | 0.329 | -0.291 | -0.086 | -0.574 |
| LEU192 | -1.831 | 0.597 | -0.356 | -0.213 | -1.802 |
| ASN193 | -1.225 | 0.282 | 0.463 | -0.193 | -0.672 |
| LEU195 | -0.212 | 0.074 | -0.307 | -0.002 | -0.447 |
| PHE196 | -2.800 | -0.062 | 1.147 | -0.473 | -2.188 |
| LYS197 | -0.091 | -11.441 | 11.620 | -0.000 | 0.087 |
| ARG199 | -0.628 | -33.285 | 31.672 | -0.227 | -2.468 |
| LEU232 | -2.006 | 0.592 | -0.030 | -0.179 | -1.622 |
| MET235 | -1.070 | -0.365 | 0.485 | -0.080 | -1.030 |
| ALA278 | -0.561 | 0.108 | -0.097 | -0.016 | -0.566 |
| LYS279 | -0.278 | -11.902 | 11.86. | -0.007 | -0.324 |
| PHE280 | -3.577 | -0.139 | 1.510 | -0.603 | -2.809 |
| ILE281 | -0.905 | -0.262 | 0.354 | -0.134 | -0.947 |

**Table.S6 Decomposition energy of key residues of Ludi-7**

|  | Vdw | Ele | Pol | Non-pol | Total |
| --- | --- | --- | --- | --- | --- |
| ALA189 | -0.782 | -0.176 | 0.527 | -0.165 | -0.596 |
| LEU192 | -1.821 | 0.957 | -0.299 | -0.237 | -1.399 |
| ASN193 | -1.944 | -0.083 | 0.919 | -0.278 | -1.386 |
| LEU195 | -0.185 | 0.584 | -0.601 | -0.001 | -0.203 |
| PHE196 | -0.309 | 0.443 | 0.542 | -0.499 | -2.553 |
| LYS197 | -0.099 | 2.399 | -2.154 | -0.000 | 0.146 |
| ARG199 | -0.733 | -14.428 | 14.265 | -0.203 | -1.099 |
| LEU232 | -1.984 | 0.817 | -0.144 | -0.182 | -1.493 |
| MET235 | -1.106 | -0.135 | 0.289 | -0.070 | -1.023 |
| ALA278 | -0.625 | 0.225 | -0.198 | -0.020 | -0.618 |
| LYS279 | -0.321 | 6.792 | -6.564 | -0.007 | -0.101 |
| PHE280 | -2.804 | 0.097 | 0.773 | -0.491 | -2.426 |
| ILE281 | -0.831 | -0.388 | 0.459 | -0.114 | -0.874 |

**Table.S7 Decomposition energy of key residues of Design-5**

|  | Vdw | Ele | Pol | Non-pol | Total |
| --- | --- | --- | --- | --- | --- |
| ALA189 | -0.386 | 0.175 | -0.196 | -0.059 | -0.466 |
| LEU192 | -1.615 | 0.459 | -0.097 | -0.195 | -1.448 |
| ASN193 | -1.214 | 0.573 | 0.465 | -0.183 | -0.359 |
| LEU195 | -0.181 | 0.043 | -0.225 | -0.001 | -0.364 |
| PHE196 | -2.081 | -0.236 | 1.163 | -0.479 | -2.353 |
| LYS197 | -0.083 | -10.673 | 10.832 | -0.000 | 0.075 |
| ARG199 | -0.742 | -30.754 | 29.934 | -0.219 | -1.782 |
| LEU232 | -2.071 | 0.761 | -0.123 | -0.192 | -1.625 |
| MET235 | -1.211 | -0.420 | 0.591 | -0.097 | -1.138 |
| ALA278 | -0.573 | 0.108 | -0.102 | -0.020 | -0.587 |
| LYS279 | -0.258 | -11.498 | 11.394 | -0.005 | -0.587 |
| PHE280 | -3.742 | -0.259 | 1.574 | -0.635 | -3.062 |
| ILE281 | -1.012 | -0.298 | 0.410 | -0.183 | -1.083 |

**Table.S8 Decomposition energy of key residues of Design-11**

|  | Vdw | Ele | Pol | Non-pol | Total |
| --- | --- | --- | --- | --- | --- |
| ALA189 | -1.159 | -0.501 | 0.761 | -0.244 | -1.144 |
| LEU192 | -2.369 | 0.404 | 0.203 | -0.181 | -1.943 |
| ASN193 | -2.115 | -0.339 | 1.428 | -0.261 | -1.287 |
| LEU195 | -0.816 | -0.108 | -0.459 | -0.033 | -1.416 |
| PHE196 | -3.225 | -0.300 | 1.147 | -0.168 | -2.846 |
| LYS197 | -0.131 | 0.056 | 0.117 | 0.000 | 0.041 |
| ARG199 | -1.285 | -1.637 | 2.633 | -0.177 | -0.467 |
| LEU232 | 0.891 | -0.192 | 0.189 | -0.084 | -0.978 |
| MET235 | -0.052 | -0.019 | 0.010 | -0.001 | -0.063 |
| ALA278 | -0.190 | -0.069 | -0.050 | -0.001 | -0.309 |
| LYS279 | -0.322 | 1.070 | -1.335 | -0.013 | -0.600 |
| PHE280 | -4.388 | -0.021 | 2.022 | -0.674 | -3.061 |
| ILE281 | -0.849 | 0.036 | 0.003 | -0.096 | -0.906 |

**Table.S9 Decomposition energy of key residues of Design-12**

|  | Vdw | Ele | Pol | Non-pol | Total |
| --- | --- | --- | --- | --- | --- |
| ALA189 | -0.787 | -0.295 | 0.744 | -0.140 | -0.478 |
| LEU192 | -2.299 | 0.233 | 0.211 | -0.189 | -2.043 |
| ASN193 | -2.023 | -0.168 | 1.144 | -0.250 | -1.297 |
| LEU195 | -0.405 | 0.165 | -0.475 | -0.036 | -0.751 |
| PHE196 | -2.638 | 0.002 | 0.781 | -0.365 | -2.220 |
| LYS197 | -0.132 | -0.056 | 0.170 | -0.001 | 0.094 |
| ARG199 | -1.163 | -3.380 | 3.595 | -0.315 | -1.264 |
| LEU232 | -1.281 | -1.096 | 0.785 | -0.182 | -1.775 |
| MET235 | -0.535 | 0.101 | -0.086 | -0.030 | -0.551 |
| ALA278 | -0.338 | -0.017 | -0.048 | -0.001 | -0.403 |
| LYS279 | -0.349 | 0.217 | -0.814 | -0.012 | -0.958 |
| PHE280 | -4.651 | -0.353 | 1.957 | -0.704 | -3.751 |
| ILE281 | -0.954 | 0.109 | 0.000 | -0.090 | -0.935 |

**Table.S10 Decomposition energy of key residues of Design-13**

|  | Vdw | Ele | Pol | Non-pol | Total |
| --- | --- | --- | --- | --- | --- |
| ALA189 | -0.946 | 0.027 | 0.340 | -0.177 | -0.757 |
| LEU192 | -2.266 | 0.700 | -0.402 | -0.195 | -2.163 |
| ASN193 | -1.789 | 0.314 | 0.486 | -0.230 | -1.219 |
| LEU195 | -0.519 | -0.176 | -0.633 | -0.023 | -1.351 |
| PHE196 | -2.577 | -0.323 | 1.060 | -0.401 | -2.242 |
| LYS197 | -0.102 | -0.624 | 0.799 | -0.000 | 0.073 |
| ARG199 | -1.164 | -4.406 | 5.020 | -0.205 | -0.755 |
| LEU232 | -1.292 | -0.518 | 0.668 | -0.126 | -1.267 |
| MET235 | -0.288 | -0.084 | 0.138 | -0.017 | -0.251 |
| ALA278 | -0.276 | -0.117 | 0.023 | -0.002 | -0.372 |
| LYS279 | -0.312 | 0.617 | -0.737 | -0.018 | -0.451 |
| PHE280 | -4.089 | -0.196 | 2.126 | -0.694 | -2.853 |
| ILE281 | -1.091 | 0.173 | -0.115 | -0.136 | -1.170 |

**Table.S11 Decomposition energy of key residues of Design-14**

|  | Vdw | Ele | Pol | Non-pol | Total |
| --- | --- | --- | --- | --- | --- |
| ALA189 | -0.049 | -0.011 | -0.029 | -0.000 | -0.090 |
| LEU192 | -0.316 | -0.088 | -0.042 | -0.013 | -0.458 |
| ASN193 | -1.767 | -1.356 | 2.322 | -0.288 | -1.089 |
| LEU195 | -0.149 | 0.002 | -0.245 | 0.000 | -0.392 |
| PHE196 | -2.887 | -0.079 | 0.767 | -0.311 | -2.510 |
| LYS197 | -1.662 | -0.756 | 1.425 | -0.227 | -1.219 |
| ARG199 | -0.382 | -0.809 | 0.865 | -0.077 | -0.403 |
| LEU232 | -0.094 | -0.030 | 0.009 | -0.005 | -0.120 |
| MET235 | -0.016 | -0.010 | 0.026 | -0.000 | -0.000 |
| ALA278 | -0.031 | -0.024 | 0.066 | -0.001 | 0.010 |
| LYS279 | -0.049 | 0.027 | 0.030 | -0.001 | 0.007 |
| PHE280 | -1.412 | -0.071 | 0.698 | -0.254 | -1.038 |
| ILE281 | -1.088 | -0.114 | 0.364 | -0.188 | -1.026 |
